# Cathepsin D interacts with adenosine A_2A_ receptors in mouse macrophages to modulate cell surface localization and inflammatory signaling

**DOI:** 10.1016/j.jbc.2022.101888

**Published:** 2022-03-31

**Authors:** Adrienn Skopál, Tamás Kéki, Péter Á. Tóth, Balázs Csóka, Balázs Koscsó, Zoltán H. Németh, Luca Antonioli, Andreas Ivessa, Francisco Ciruela, László Virág, György Haskó, Endre Kókai

**Affiliations:** 1 Department of Medical Chemistry, Faculty of Medicine, University of Debrecen, Debrecen, Hungary; 2 Doctoral School of Molecular Medicine, University of Debrecen, Debrecen, Hungary; 3 Department of Anesthesiology, Columbia University, New York, New York, USA; 4 Department of Pathology, University of Massachusetts Medical School, Worcester, Massachusetts, USA; 5 Department of Surgery, Morristown Medical Center, Morristown, New Jersey, USA; 6 Department of Clinical and Experimental Medicine, University of Pisa, Pisa, Italy; 7 Department of Cell Biology and Molecular Medicine, Cardiovascular Research Institute, Rutgers New Jersey Medical School, Newark, New Jersey, USA; 8 Pharmacology Unit, Department of Pathology and Experimental Therapeutics, School of Medicine and Health Sciences, Institute of Neurosciences, University of Barcelona, L'Hospitalet de Llobregat, Barcelona, Spain; 9 Neuropharmacology and Pain Group, Neuroscience Program, Bellvitge Institute for Biomedical Research, L'Hospitalet de Llobregat, Barcelona, Spain; 10 MTA-DE Cell Biology and Signaling Research Group, University of Debrecen, Debrecen, Hungary

**Keywords:** adenosine receptor, cathepsin D, protein interaction, macrophage, inflammation, AA, amino acid, A_1_R, adenosine A_1_ receptor, A_2A_R, adenosine A_2A_ receptor, A_2B_R, adenosine A_2B_ receptor, A_3_R, adenosine A_3_ receptor, BCA, bicinchoninic acid, BSA, bovine serum albumin, cDNA, complementary DNA, CDS, coding sequence, co-IP, coimmunoprecipitation, CtsD, cathepsin D, DAPI, 4′,6-diamidino-2-phenylindole, DMEM, Dulbecco's modified Eagle's medium, DNA-BD, DNA-binding domain, FBS, fetal bovine serum, GAL4 DNA-BD, GAL4 DNA-binding domain, GPCR, G protein–coupled receptor, GRK, G protein–coupled receptor kinase, GST, glutathione-*S*-transferase, HC, heavy chain, HEK-293, human embryonic kidney-293 cell line, IgG, immunoglobulin G, IL, interleukin, IPMΦ, mouse peritoneal macrophage, IS, immunostaining, LAS X, Leica Application Suite X, LPS, lipopolysaccharide, NA, numerical aperture, pCMV, plasmid cytomegalovirus, PD, pull-down, PIC, Protease Inhibitor Cocktail, RIPA, radioimmunoprecipitation assay, WB, Western blot, YTH, yeast two-hybrid assay

## Abstract

Adenosine A_2A_ receptor (A_2A_R)–dependent signaling in macrophages plays a key role in the regulation of inflammation. However, the processes regulating A_2A_R targeting to the cell surface and degradation in macrophages are incompletely understood. For example, the C-terminal domain of the A_2A_R and proteins interacting with it are known to regulate receptor recycling, although it is unclear what role potential A_2A_R-interacting partners have in macrophages. Here, we aimed to identify A_2A_R-interacting partners in macrophages that may effect receptor trafficking and activity. To this end, we performed a yeast two-hybrid screen using the C-terminal tail of A_2A_R as the “bait” and a macrophage expression library as the “prey.” We found that the lysosomal protease cathepsin D (CtsD) was a robust hit. The A_2A_R–CtsD interaction was validated *in vitro* and in cellular models, including RAW 264.7 and mouse peritoneal macrophage (IPMΦ) cells. We also demonstrated that the A_2A_R is a substrate of CtsD and that the blockade of CtsD activity increases the density and cell surface targeting of A_2A_R in macrophages. Conversely, we demonstrate that A_2A_R activation prompts the maturation and enzymatic activity of CtsD in macrophages. In summary, we conclude that CtsD is a novel A_2A_R-interacting partner and thus describe molecular and functional interplay that may be crucial for adenosine-mediated macrophage regulation in inflammatory processes.

The endogenous nucleoside adenosine plays an essential role in the regulation of homeostasis in response to stressful situations, such as low energy supply or cell damage. Extracellular adenosine is generated locally in tissues under conditions of hypoxia, ischemia, inflammation, and trauma (reviewed in Ref. (1)). Extracellular adenosine–mediated signaling is achieved by four G protein–coupled receptors (GPCRs), namely adenosine A_1_, A_2A_, A_2B_, and A_3_ receptors (*i.e.*, A_1_R, A_2A_R, A_2B_R, and A_3_R, respectively) (2). While the A_1_R, A_2A_R, and A_2B_R have a primary protein structure that is highly conserved during evolution, A_3_Rs exhibit significant sequence variations among species (3). Conversely, and in contrast to the other three adenosine receptors, both the human and mouse A_2A_R have a long intracellular carboxyl terminus tail comprising 122 amino acids in humans (UniProtKB/Swiss-Prot no.: P29274.2) and 120 amino acids in mice (UniProtKB/Swiss-Prot no.: Q60613.3). The A_2A_R crystal structure was the first to be determined among adenosine receptors (4), and up to now, several agonist- and antagonist-binding structural models already exist (5, 6). However, these models do not provide information about the C-terminal domain. Functional studies of the A_2A_R demonstrated that the juxtamembrane part of the C-terminal domain, following the seventh transmembrane helix, contributes to the appropriate folding, ligand binding, and signaling of the receptor (7).

The C-terminally truncated A_2A_R (1–309 from dog) can bind to agonist and antagonist ligands, but its affinities are reduced (8). The absence of the entire A_2A_R C terminus (*i.e.*, 1–311 human A_2A_R form) has divergent effects on cAMP accumulation and mitogen-activated protein kinase activation when expressed in human embryonic kidney-293 (HEK-293) cells (3, 9). As recently demonstrated, the removal of the last 40 amino acids from the C-terminal region of the A_2A_R resulted in weaker agonist-binding affinity and significantly decreased antagonist-binding affinity (10). Furthermore, Robinson *et al.* (11) demonstrated that the truncated human A_2A_R (A_2A_R^Δ316R^) does not activate the cAMP signaling pathway, and the C-terminal tail is not important for Gα specificity. Finally, it is important to note that the A_2A_R C-terminal tail may show high lateral flexibility as it lacks a putative palmitoylation site (12). Thus, a cysteine residue at the end of helix 8 is responsible for anchoring A_1_R, A_2B_R, and A_3_R to the plasma cell membrane, whereas the A_2A_R lacks this cysteine residue (13).

The structural features of A_2A_R, together with the large number of identified partner proteins so far (14, 15, 16, 17, 18, 19, 20, 21, 22, 23, 24, 25), indicate that the relatively long, motile, mainly unfolded (21), C-terminal tail of this receptor is adapted to serve as a binding hub. A_2A_R C-terminal domain–binding proteins are responsible for the following functions: (i) anchoring the receptor to the actin cytoskeleton and regulating receptor recycling (14), (ii) regulating the mitogen-activated protein kinase signaling pathway (15, 17), (iii) triggering crosstalk with other transmembrane receptors (16), and (iv) modulating receptor activity and exiting the endoplasmic reticulum (18, 19, 20).

Cathepsin D (CtsD) is an aspartyl protease that is activated by low pH in lysosomes, leading to the degradation of phagocytosed extracellular proteins (22). CtsD is synthesized as an inactive enzyme. Transport through the *trans*-Golgi network and delivery to the lysosomes *via* endosomes is dependent on N-linked glycosylation and phosphorylation on mannose residues. In acidic vesicles, the proform of CtsD single chain undergoes several cleavage steps. First, an active intermediate is generated by cysteine proteases, and then, this single-chain molecule is cleaved by cathepsin L and cathepsin B into the fully active N-terminal light chains and C-terminal heavy chains (HCs) (26). About 90% of CtsD in the lysosomes and endosomes is soluble, whereas 10% of the enzyme is membrane bound (27). CtsD is secreted into the extracellular space, and it is also released from the lysosome into the cytoplasm. The dual localization of CtsD accounts for its participation in various physiological processes, including activation of different hormones and enzymes, processing of antigens, and regulation of apoptosis (reviewed by Dubey and Luqman (28)). In macrophages, the expression level of CtsD is elevated compared with other cell types, and it is associated with endosomal membranes (29). Here, we report for the first time that CtsD binds to the A_2A_R C-terminal domain in mouse macrophages. We demonstrate that CtsD proteolytically degrades the A_2A_R, thus regulating the expression of the receptor in mouse macrophages. Conversely, we provide evidence that A_2A_R activation increases the maturation and enzymatic activity of CtsD in macrophages.

## Results

### Generation of a complementary DNA library from mouse peritoneal macrophages

To identify A_2A_R-interacting proteins in mouse macrophages by yeast two-hybrid (YTH) assay, we constructed a complementary DNA (cDNA) library from mouse peritoneal macrophages (IPMΦ) using the SMART cDNA Library Construction Kit. High yields of double-stranded cDNA were generated from 2 μg of total mouse RNA. After nucleospin purification and enrichment, we isolated an IPMΦ cDNA pool and used control mouse liver cDNA pool (Fig. S1*A*). To determine the quality of the newly synthesized IPMΦ and control cDNA libraries, we used these libraries as templates for PCR amplification of six different genes: Adora1, Adora2a, and Adora2b, F4/80 receptor, tumor necrosis factor α (TNFα), and peroxisome proliferator–activated receptor γ (PPARγ). We detected these specific DNA products in both cDNA libraries and all six amplification reactions (Fig. S1*B*). We then cotransformed the IPMΦ cDNA pool successfully with the pGADT7-Rec “prey” vector into the *Saccharomyces cerevisiae* Y187 strain (Fig. S1*C*). Therefore, the “prey” cDNA library vector containing yeast strain was suitable for YTH screening.

### Identification of CtsD as an A_2A_R-interacting protein

The C terminus of the mouse A_2A_R (GenBank accession no.: NM_009630) was used as a bait in YTH system for screening our prey mouse IPMΦ cDNA library. To this end, we first transformed yeast cells (Y2H Gold strain) with the “bait” vector (*i.e.*, pGBKT7 plasmid containing Adora2a^284–410^) encoding the A_2A_R C-terminal tail fused to GAL4 DNA-binding domain (GAL4 DNA-BD). The transformed yeast cells (Y2H Gold strain) expressed the C-terminal 126 amino acid residues of mouse A_2A_R (A_2A_R^284–410^) with the GAL4 DNA-BD as a fusion protein. The cMyc epitope tag allowed us to detect the expression of the fusion protein in *S. cerevisiae* Y2H Gold yeast cells by immunoblot (Fig. S2*A*). Thus, the cMyc-specific antibody recognized a single band with a molecular weight of 36 kDa in the lysate of the Y2H Gold yeast cells after transformation with the pGBKT7-A_2A_R^284–410^ plasmid construct. Similarly, we also checked the expression of BD-cMyc-p53 and BD-cMyc-Lamin fusion proteins, which were used as control “baits.” After we constructed and tested our cDNA library from mouse IPMΦ cells with the “prey” pGADT7 vector (Fig. S1, *A* and *B*), we transformed the cDNA library into the yeast Y187 strain. Next, the haploid Y2H Gold and Y187 cells containing the “bait” and “prey” library vectors, respectively, were cocultured to mate and create diploid cells. Diploid cells contained the four reporter genes (*AUR1-C*, *ADE2*, *HIS3*, and *MEL1*) that were activated in response to YTH interactions on selective media (Fig. S2*B*). Accordingly, 4.6 × 10^9^ yeast colonies were screened and 23 positive clones were identified, 15 of which encoded CtsD.

Since the suppressive effect of adenosine on the production of proinflammatory and stimulatory effects on anti-inflammatory cytokines in macrophages is mediated predominantly by A_2A_R (30, 31, 32), we presumed that the library was suitable for detecting physiologically relevant interactions.

### Validation of the interaction between CtsD and the A_2A_R C-terminal tail in yeast

**Figure 1:**
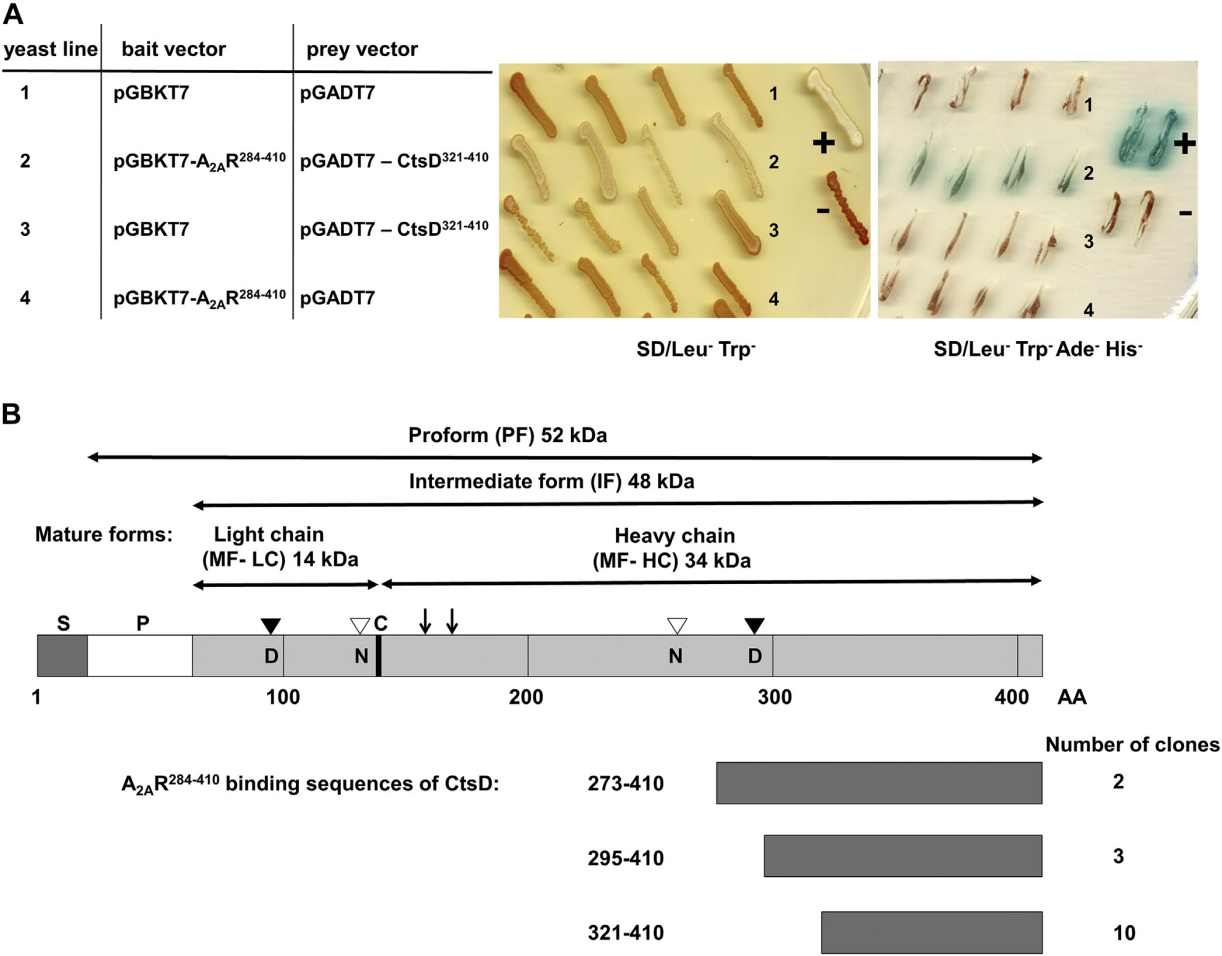
**Validation of the interaction between CtsD and the A**_**2A**_**R C-terminal tail in yeast.***A*, transformation of Yeast 2H Gold strain with pGBKT7, pGBKT7-A_2A_R^284–410^ (bait), pGADT7 and pGADT7-CtsD^321–410^ (prey) vectors in a pairwise combination, according to the numbered yeast line. The yeast cells were grown on SD/Leu^−^ Trp^−^ or SD/Leu^−^ Trp^−^ Ade^−^ His^−^ (in the presence of Aureobasidin and X-α-Gal) selective medium. pGBKT7-p53 and pGADT7-SV40 small T antigen were used as positive controls and pGBKT7-p53 and pGADT7-Lamin expressing vectors were used as negative controls. *B*, presentation of the different maturation forms of CtsD. Characterization of A_2A_R^284–410^ interactor clones that encode the different ORFs of the CtsD. The amino acid numbers of pre–pro-CtsD protein (P18242) is based on UniProtKB/Swiss-Prot databases. S: N-terminal secretion signal peptide (20 amino acids [AA]); P: propeptide (44 AA); C: cleavage site between the light and heavy chain of CtsD; *arrows* indicate the processing sites; D: catalytic Asp residues (D97 and D295) are depicted by *black triangles*; N: N-linked glycosylation sites at Asn residues (N134 and N261) are depicted by *empty triangles* (26). A_2A_R, adenosine A_2A_ receptor; CtsD, cathepsin D.

The interaction between A2_A_^284–410^ and CtsD^321–410^ was further validated by yeast retransformation in four independent colonies (Fig. 1*A*). The specificity of the binding was confirmed on selective media, where the positive yeast colonies complement the absence of four different reporter genes and activate the *MEL1* gene that encodes alpha-galactosidase enzyme, which hydrolyzes its chromogenic substrate, X-alpha-Gal, yielding a blue precipitate. The identified CtsD interactor clones included ten cDNAs encoding the C-terminal 89 amino acids (AA321–410), three cDNAs encoding 115 amino acids (AA295–410), and two cDNAs encoding 137 amino acids (AA273–410) of CtsD (Fig. 1*B*). Interestingly, the three different groups of positive clones with overlapping sequences indicated that this high-frequency hit was not false positive because of the overamplification of a particular CtsD segment in the library. These data suggested a strong interaction between C-terminal tail of A_2A_R and CtsD. The interacting CtsD sequences were mapped onto the primary sequence of mouse pre-pro-CtsD protein (P18242), and amino acid sequence of CtsD was numbered according to the UniProtKB/Swiss-Prot database (Fig. 1*B*).

### Validation of the A_2A_R C-terminal tail and CtsD interaction in macrophages

**Figure 2:**
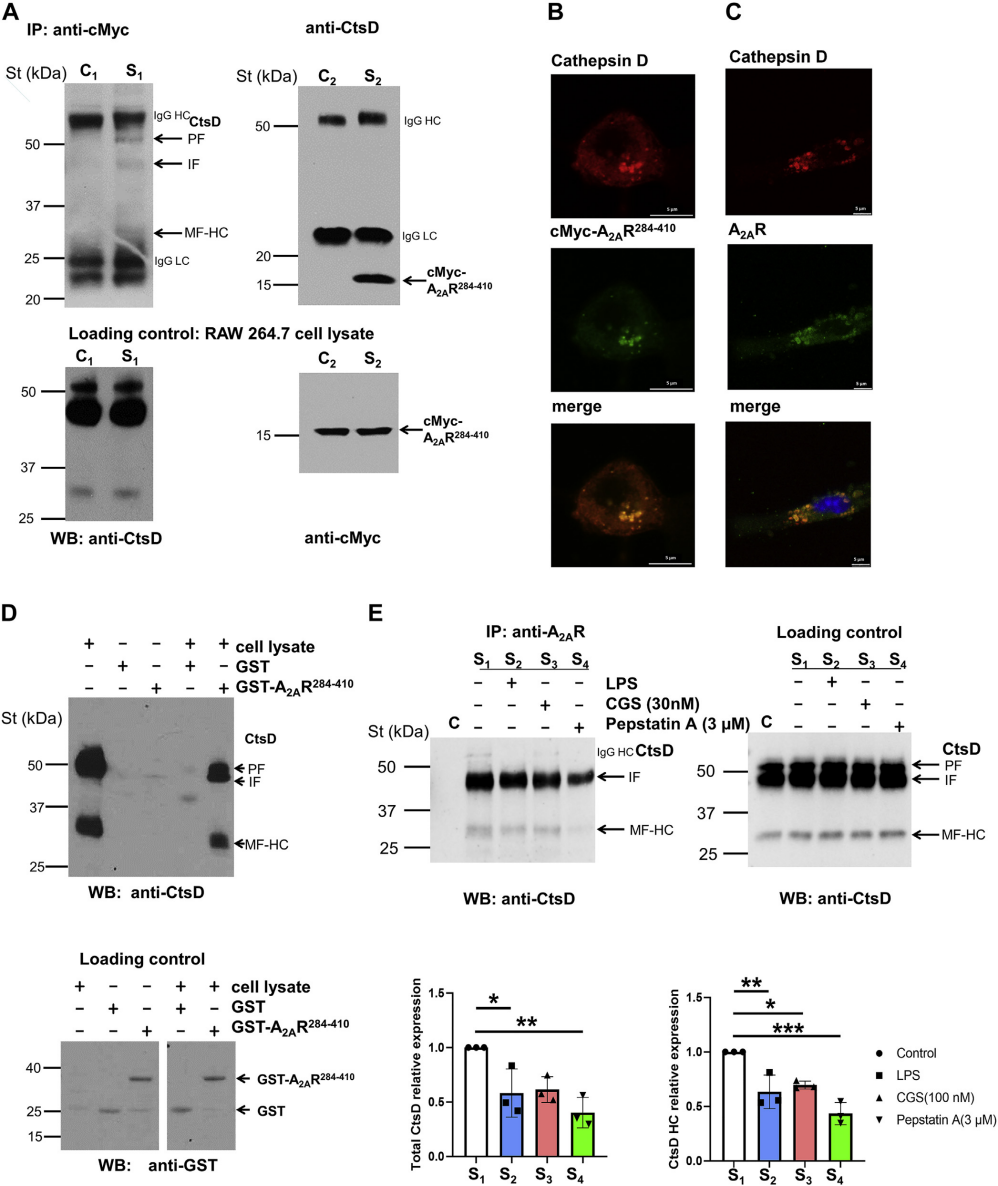
**Validation of the interaction between CtsD and the A**_**2A**_**R in macrophages.***A*, RAW 264.7 cells were transfected with a pCMV-cMyc-A_2A_R^284–410^ construct. The recombinant cMyc-A_2A_R^284–410^ and the endogenous CtsD were coimmunoprecipitated in RAW 264.7 cells. Specific bands were detected in the immune complex by WB using cMyc and CtsD antibodies. The IP was carried out using 10 μg of specific antibody in the sample (S_1_ and S_2_) and the same amount of isotype control IgG_1_ (C_1_) and goat control serum (C_2_) in the control experiments. For loading controls (*lower panel*), 10 μg of total protein were analyzed in each lane. CtsD PF, proform; IF, intermediate form; MF-HC, mature form of heavy chain; St denotes Precision Plus Protein Dual Color Standards; and IgG HC, LC denotes immunoglobulin heavy and light chains, respectively. *B*, colocalization of cMyc-A_2A_R^284–410^ and endogenous CtsD in RAW 264.7 cells by IS. CtsD-specific and cMyc-specific primary antibodies were used to identify target proteins. The secondary antibodies were labeled with Alexa-647 (*red*) and Alexa-546 (*green*), respectively, and specific labeling was visualized by confocal microscopy (Leica TCS SP8). *C*, colocalization of endogenous A_2A_R and CtsD was detected in mouse IPMΦ cells by IS. CtsD-specific and A_2A_R-specific primary antibodies were used for the identification of target proteins. The secondary antibodies were labeled with Alexa-647 (*red*) and Alexa-546 (*green*), respectively, and the nucleus was stained with DAPI. The images were captured by confocal microscopy (Leica TCS SP8). *D*, endogenous CtsD was pulled down by recombinant GST-A_2A_R^284–410^ but not by GST alone in the cell lysate of IPMΦs. In the cell lysate, 10 μg of total protein were incubated with GST and GST-A_2A_R^284–410^ recombinant proteins, and the binding of CtsD was detected using CtsD-specific antibody. GST or GST-A_2A_R^284–410^ recombinant proteins in each lane were detected with GST-specific antibodies (*lower panel*). CtsD PF, proform; IF, intermediate form; MF-HC, mature form of heavy chain; St denotes Precision Plus Protein Dual Color Standards. *E*, the endogenous A_2A_R and the CtsD were coimmunoprecipitated in RAW 264.7 cells. Specific bands were detected in the immune complex by WB using CtsD antibodies. The IP was carried out using 8.5 μg of A_2A_R-specific antibody in the sample (S_1_–S_4_), and antibody was not added in the control experiment (*C*). For loading controls (*right panel*), 10 μg of total protein were analyzed in each lane. CtsD PF, proform; IF, intermediate form; MF-HC, mature form of heavy chain; St denotes Precision Plus Protein Dual Color Standards; IgG HC, immunoglobulin heavy chain, respectively. Densitometry analysis data are presented as mean ± SD of three independent experiments. Values from ANOVA: *F* = 9.217 and *p* = 0.0057 for total CtsD and *F* = 18.59 and *p* = 0.0006 for CtsD HC. ∗*p* < 0.05, ∗∗*p* < 0.01, and ∗∗∗*p* < 0.001 *versus* control (in the absence of A_2A_R-specific antibody). A_2A_R, adenosine A_2A_ receptor; CtsD, cathepsin D; DAPI, 4′,6-diamidino-2-phenylindole; GST, glutathione-*S*-transferase; IP, immunoprecipitation; IPMΦ, mouse peritoneal macrophage; IS, immunostaining; pCMV, plasmid cytomegalovirus; WB, Western blot.

To determine whether the interaction between A_2A_R and CtsD may occur endogenously in higher eukaryotes, we designed a coimmunoprecipitation (co-IP) experiment in the RAW 264.7 mouse macrophage cell line. To this end, we transfected the C terminus of A_2A_R tagged with the cMyc epitope (*i.e.*, cMyc-A_2A_R^284–410^) into RAW 264.7 cells. Interestingly, in transfected RAW 264.7 cells, the anti-cMyc antibody was able to immunoprecipitate the pro-CtsD, intermediate CtsD, and mature heavy-chain CtsD forms from soluble extracts, as detected by immunoblotting using an anti-CtsD–specific antibody (Fig. 2*A*, *left panel*). Importantly, these CtsD protein bands were not observed when a control isotype antibody was used in the co-IP. The anti-CtsD antibody detected the same bands in the control and the sample cell lysate extracts used in the co-IP (Fig. 2*A*, *left panel*). Similarly, the anti-CtsD antibody was able to immunoprecipitate the cMyc-A_2A_R^284–410^ from the same soluble extracts, as an immunoreactive band of 18 kDa was detected when an anti-cMyc–specific antibody was used in the immunoblot (Fig. 2*A*, *right panel*). Interestingly, this cMyc-A_2A_R^284–410^ protein band was not detected when a control isotype antibody was used in the co-IP. The anti-cMyc antibody detected the same 18 kDa band in both cell lysate extracts used in the co-IP (Fig. 2*A*, *right panel*). Next, we assessed the codistribution of the A_2A_R C-terminal tail and CtsD by immunocytochemistry experiments. Interestingly, the C-terminal domain of A_2A_R and the endogenous CtsD showed a high degree of codistribution (Pearson coefficient of 0.78; Fig. 2*B*).

To study the interaction in primary cells, we expressed and purified the C terminus of the A_2A_R as a glutathione-*S*-transferase (GST) fusion protein in the *Escherichia coli* BLR strain and used this fusion protein as a “bait” for a “pull-down” (PD) experiment in IPMΦ cells. The anti-CtsD antibody was able to detect the pro-CtsD, intermediate CtsD, and mature HC CtsD forms in the GST-A_2A_R^284–410^–specific complex from the soluble IPMΦ cell lysate (Fig. 2*D*). Importantly, these CtsD protein bands were not observed when a control GST protein was used as a “bait” in the PD experiment. The anti-GST antibody detected the same GST and GST-A_2A_R^284–410^–specific bands, respectively, in both cell lysate extracts used in the PD (Fig. 2*D*). Endogenous A_2A_R and CtsD colocalized in IPMΦs as determined using immunocytochemistry. That is, endogenous A_2A_R and CtsD showed a high degree of codistribution (Pearson coefficient of 0.81, Fig. 2*C*). Overall, these results further validate A_2A_R–CtsD interactions in macrophages.

We then assessed whether the endogenous A_2A_R and CtsD interact in RAW 264.7 cells by co-IP experiment. Indeed, an anti-A_2A_R antibody was able to immunoprecipitate the intermediate CtsD and mature HC CtsD forms from soluble RAW 264.7 extracts, as detected by immunoblotting using an anti-CtsD–specific antibody (Fig. 2*E*, *left panel*). Importantly, these CtsD protein bands were not observed in the absence of the anti-A_2A_R antibody in the co-IP experiment. Thus, these results demonstrated that endogenous A_2A_R and CtsD tonically interact in RAW 264.7 cells, thus highlighting the importance of this interaction in the biology of these two proteins. We next investigated the impact of either lipopolysaccharide (LPS)-mediated inflammatory stimulation, and A_2A_R activation and aspartyl protease inhibition in the A_2A_R–CtsD interaction was also assessed through co-IP experiments. Interestingly, the treatment of RAW 264.7 cells with LPS and aspartyl protease inhibitor significantly reduced the interaction of the receptor with both the intermediate and mature forms of CtsD (Fig. 2*E*, *bottom panel* S_2_ and S_4_, respectively). Instead, the incubation of the cells with CGS21680, an A_2A_R-specific agonist, only affected the interaction of the receptor with the CtsD HC form (Fig. 2*E*, *lower panel* and Fig. S3). Overall, these results suggested that the A_2A_R–CtsD interaction depends on the inflammatory environment of macrophages.

### A_2A_R is a putative substrate of CtsD protease

**Figure 3:**
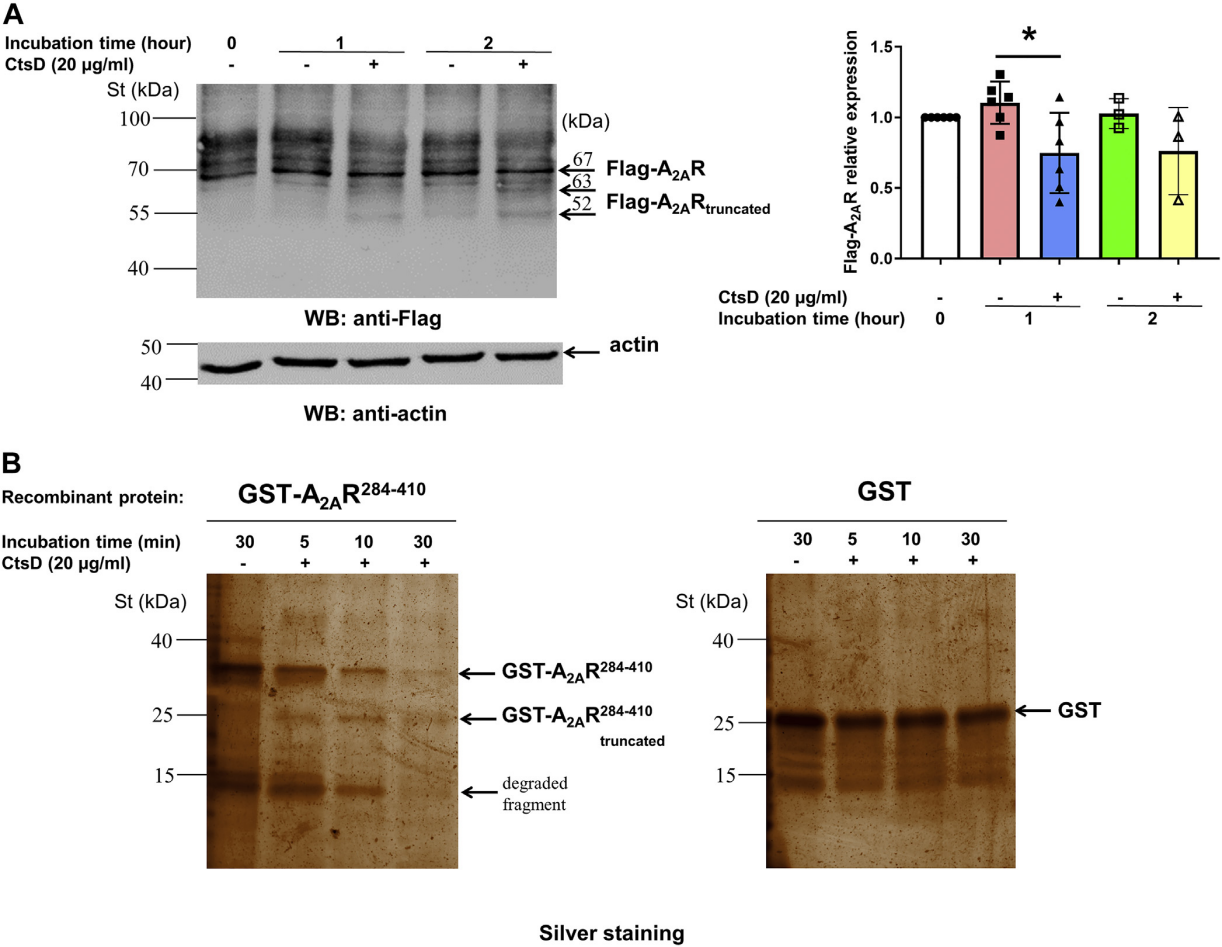
**The A**_**2A**_**R is a potential substrate of CtsD.***A*, lysates from HEK-293-FLAG-A_2A_R^SNAP^ cells were incubated in the absence and presence of recombinant mouse CtsD (1.5 μg/ml) for 1 and 2 h. The specific bands were detected by WB using FLAG-tag and β-actin-specific antibodies. Densitometry analysis data are presented as mean ± SD of three independent experiments. Values from ANOVA: *F* = 3.385 and *p* = 0.0298. ∗*p* < 0.05 *versus* control (in the absence of recombinant mouse CtsD). *B*, 200 ng of recombinant GST-A_2A_R^284–410^ and GST proteins were incubated in the absence and presence of recombinant mouse CtsD (10 μg/ml) for 5, 10, and 30 min. The bands of the recombinant proteins were detected in polyacrylamide gel by silver staining. St denotes Precision Plus Protein Dual Color Standards. A_2A_R, adenosine A_2A_ receptor; CtsD, cathepsin D; GST, glutathione-*S*-transferase; HEK-293, human embryonic kidney 293 cell line; WB, Western blot.

Once the A_2A_R–CtsD interaction was demonstrated *in vitro* and in living cells, we aimed to assess whether this physical interaction of A_2A_R and CtsD has any functional consequences. Accordingly, we next studied whether CtsD can directly cleave the A_2A_R. To this end, we first analyzed the primary protein sequence of the receptor by three different Expasy-based protease cleavage site prediction methods (https://web.expasy.org/peptide_cutter, https://prosper.erc.monash.edu.au, and https:/www.dmbr.ugent.be/prx/bioit2-public/SitePrediction). Interestingly, the algorithms used identified several CtsD cleavage sites within the A_2A_R, but only three peptide sequences (AA37–48, AA81–97, and AA371–377) were coincidently identified by all three methods. Interestingly, the AA371 to 377 peptide sequence is located within the C-terminal region of the receptor used as a bait for YTH screen, co-IP, and PD methods. Thus, this computational result would be compatible with a functional (*i.e.*, catalytic) interaction of CtsD with A_2A_R. Accordingly, we next aimed at demonstrating the direct CtsD cleavage of A_2A_R. To this end, we incubated cell extracts from HEK-293 cells permanently expressing FLAG-A_2A_R^SNAP^ with recombinant mouse CtsD (1.5 μg/ml) for 1 or 2 h (Fig. 3*A*). Immunoblot detection of FLAG-A_2A_R^SNAP^ permanently expressed in HEK-293 cells revealed a major protein band of ∼67 kDa corresponding to the expected molecular weight for A_2A_R (*i.e.*, ∼48 kDa) plus the SNAP tag protein (*i.e.*, ∼19 kDa). In addition, higher molecular weight protein bands, likely corresponding to FLAG-A_2A_R^SNAP^ glycosylated forms, were observed, as previously reported (14). Importantly, CtsD treatment significantly reduced the density of the full-length FLAG-A_2A_R^SNAP^ (*i.e.*, ∼67 kDa) from cell extracts after 1 h of incubation (Fig. 3*A*). Interestingly, in addition to the full-length FLAG-A_2A_R^SNAP^ band, two smaller molecular size bands (∼63 and ∼52 kDa) were also detected by the anti-FLAG antibody (Fig. 3*A*). Interestingly, detection of the ∼63 kDa A_2A_R protein band fits with the result of a putative CtsD cleavage of the receptor at the computationally predicted site (*i.e.*, AA371–377), thus generating an A_2A_R lacking the last 33 to 39 AA. However, the appearance of the ∼52 kDa A_2A_R protein band is not expected based on the do not match with any of the predicted CtsD cleavage sites, thus suggesting the existence of additional CtsD-sensitive proteolytic sites, not yet predicted, within the A_2A_R C-terminal domain. Finally, to further confirm the ability of CtsD to cleave the C-terminal A_2A_R domain, we incubated the recombinant GST-A_2A_R^284–410^ with the CtsD enzyme (10 μg/ml) for 5, 10, and 30 min. Interestingly, CtsD reduced time dependently the intensity of GST-A_2A_R^284–410^ protein band (Fig. 3*B*), with the concomitant production of a truncated form of GST-A_2A_R^284–410^, thus likely missing the last 33 to 39 AA. In addition, CtsD also degraded the 14 kDa degradation product generated during the GST-A_2A_R^284–410^ purification (Fig. 3*B*). Overall, these results demonstrated that A_2A_R is a substrate of CtsD.

### An aspartyl protease–specific inhibitor increases A_2A_R expression in mouse macrophages

We next examined the effect of blocking CtsD activity on the expression of A_2A_R in mouse IPMΦ cells. To this end, we incubated the cells with the oligopeptide-conjugated Pepstatin A penetratin (RQIKIWFQNRRMKWKK [pAntp(43–58)]), a potent inhibitor of aspartyl proteases (33), whereas the A_2A_R expression and subcellular localization were monitored by immunofluorescence followed by high content confocal microscopy analysis. We treated IPMΦ cells in the absence or the presence of LPS as to increase A_2A_R expression in macrophages, as previously described (34, 35).

**Figure 4:**
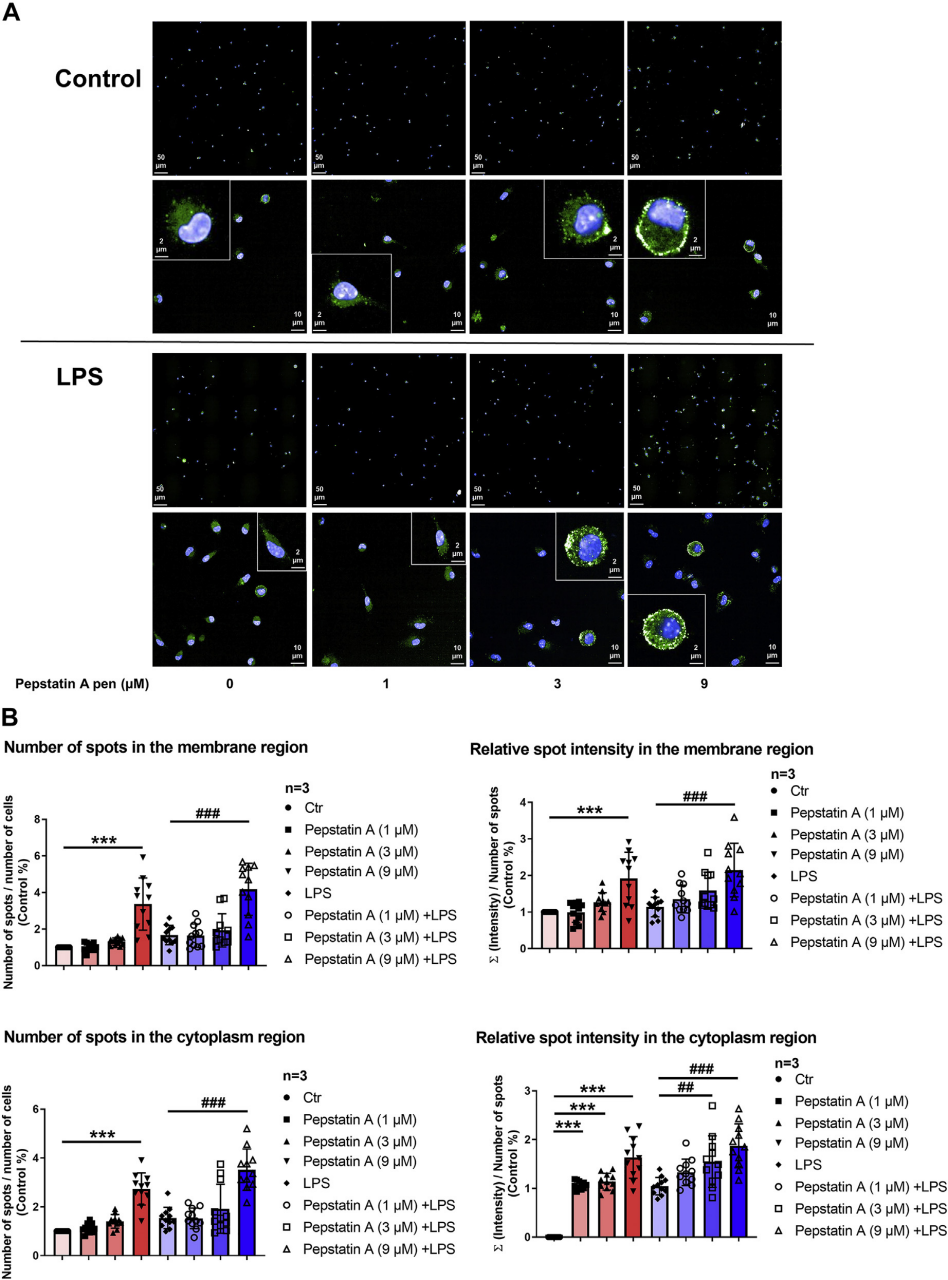
**Pepstatin A penetratin increases A**_**2A**_**R expression in mouse macrophages.** IPMΦ cells were treated with Pepstatin A penetratin (1, 3, and 9 μM) in the absence or presence of LPS (100 ng/ml) for 4 h. *A*, immunofluorescence detection of A_2A_R in IPMΦ cells. A_2A_R was stained using a specific primary antibody, and Alexa-488–conjugated secondary antibody demonstrates the localization of endogenous A_2A_R protein (*green*). Nuclei of macrophages were stained with DAPI (*blue*). Images were acquired using the Opera Phenix High Content Confocal System (PerkinElmer); 190 fields and 2000 to 3000 cells were acquired per well, and laser-based autofocus was performed for each imaging position. Images of DAPI and Alexa-488 channels were collected at 2 μm of Z image plane using a 63× water immersion objective (NA, 1.15) to visualize the cells and the localization of A_2A_R. *B*, the primary data were analyzed using Harmony 4.8 software (PerkinElmer). Spot Analyses Ready to Made Solution (http://www.perkinelmer.com/product/harmony-4-2-office-hh17000001) was used with custom modifications. Image intensities were rescaled, and cells were identified using the DAPI signal. Cellular phenotypes were characterized based on the Alexa-488 signal. Cellular features, such as the number of spots, relative spot intensity in the membrane, and cytoplasm regions, were extracted. Statistical analyses of parallel datasets were made using GraphPad Prism 8 program. The evaluation of the data based on the individual analyses of 2000 to 3000 different cells is presented as mean ± SD. Values from ANOVA: *F* = 20.53 and *p* < 0.001, *F* = 24.78 and *p* < 0.001 for number of spots in membrane, cytoplasm region, respectively; *F* = 9.52 and *p* < 0.001, *F* = 35.28 and *p* < 0.001 for relative spot intensity in membrane, cytoplasm region, respectively. ∗∗*p* < 0.01 and ∗∗∗*p* < 0.001 *versus* control (vehicle treated); ^##^*p* < 0.01, ^###^*p* < 0.001 *versus* LPS-treated cells. A_2A_R, adenosine A_2A_ receptor; DAPI, 4′,6-diamidino-2-phenylindole; IPMΦ, mouse peritoneal macrophage; LPS, lipopolysaccharide; NA, numerical aperture.

Interestingly, our results demonstrated that A_2A_R localized mainly to the plasma membrane and cytoplasm (Fig. 4*A*). Hereafter, confocal images revealed that Pepstatin A penetratin significantly increased the number, size, and intensity of A_2A_R-specific fluorescent spots in a concentration-dependent manner in both LPS-activated and control IPMΦs, both in the plasma membrane and cytoplasm (Figs. 4*B* and S3). These results are consistent with our earlier findings that the A_2A_R is a substrate for CtsD (Fig. 3).

### Pepstatin A penetratin modulates cytokine production in LPS-activated IPMΦ cells

**Figure 5:**
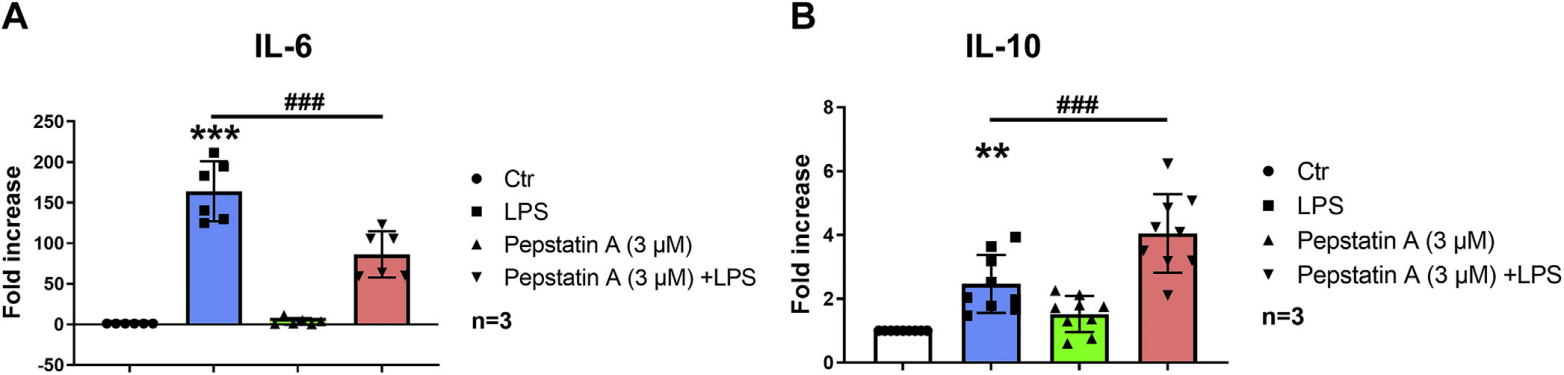
**Pepstatin A penetratin, an aspartyl protease–specific inhibitor, modifies A**_**2A**_**R-mediated cytokine production in LPS-activated macrophages.***A*, IL-1 and *B*, IL-6 in Pepstatin A penetratin treated IPMΦ cells after 4 h of LPS activation. Cytokine levels were determined using ELISA. Data are presented as mean ± SD of three independent experiments. Values from ANOVA: *F* = 66.34 and *p* < 0.001 for IL-6 and *F* = 24.44 and *p* < 0.001 for IL-10. ∗∗*p* < 0.01 and ∗∗∗*p* < 0.001 *versus* control (vehicle treated); ^##^*p* < 0.01, ^###^*p* < 0.001 *versus* LPS-treated cells. A_2A_R, adenosine A_2A_ receptor; IL, interleukin; IPMΦ, mouse peritoneal macrophage; LPS, lipopolysaccharide.

Macrophages have a prominent function in inflammation. The hallmark of this physiological process is the regulation of inflammatory and anti-inflammatory cytokine production. Since A_2A_R stimulation decreases interleukin 6 (IL-6) secretion and increases IL-10 release by macrophages (36, 37, 38), we examined whether inhibiting aspartyl proteases, which increase A_2A_R expression in IPMΦs (Fig. 4), can mimic A_2A_R stimulation. To this end, we measured the release of IL-6 and IL-10 in IPMΦ cells. Pepstatin A penetratin treatment reduced IL-6 (0.6-fold) and increased IL-10 (1.68-fold) release in LPS-activated IPMΦ cells (Fig. 5). This indicates that Pepstatin A penetratin can mimic the effect of A_2A_R stimulation.

### A_2A_R activation increases the maturation and enzymatic activity of CtsD in activated macrophages

**Figure 6:**
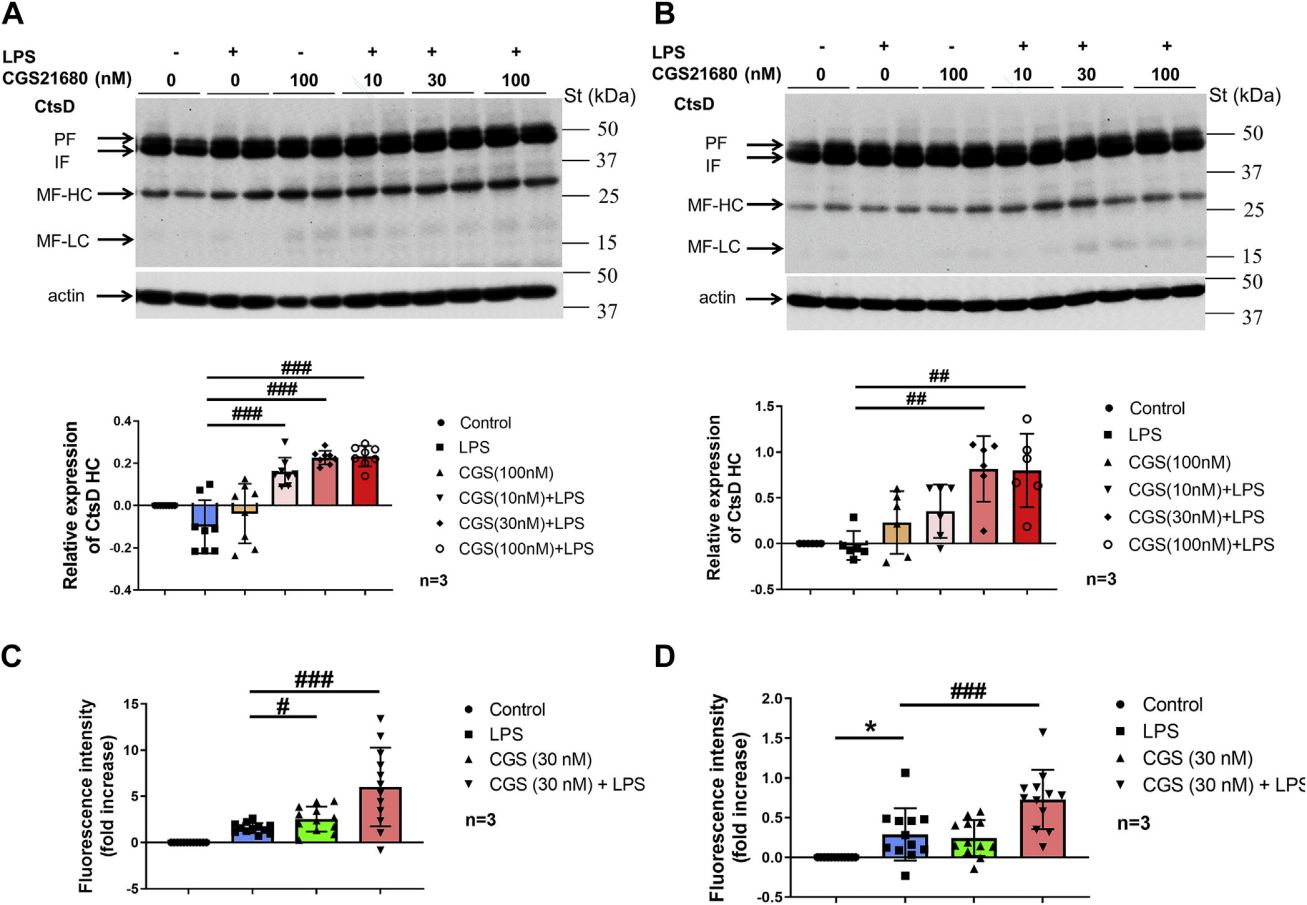
**A**_**2A**_**R activation increases the maturation and enzyme activity of CtsD in macrophages.***A*, protein samples were isolated from RAW 264.7 cells after LPS activation and treatment with the A_2A_R agonist CGS21680. *B*, protein samples were isolated from mouse IPMΦ cells after the same treatment as in *A*. About 10 μg of total protein sample in each lane were analyzed in duplicate by WB using CtsD-specific polyclonal antibody. Sample loading was normalized for β-actin. Statistical analyses of the relative expression of CtsD heavy chain (HC) are based on three independent experiments. Data are presented as mean ± SD. Values from ANOVA: *F* = 9.641 and *p* < 0.001 for IPMΦ and *F* = 22.91 and *p* < 0.001 for RAW 264.7 cells. ∗∗*p* < 0.01, ∗∗∗*p* < 0.001 *versus* LPS-activated cells. *C*, secreted and *D*, cytosolic aspartyl protease–specific activity of A_2A_R agonist-treated LPS-activated IPMΦ cells. Statistical evaluation of aspartyl protease–specific activity is based on three independent experiments. Data are presented as mean ± SD. Values from ANOVA: *F* = 15.34 and *p* < 0.001 for secreted and *F* = 14.74 and *p* < 0.001 for cytosolic aspartyl protease–specific activity. ∗*p* < 0.05; ∗∗∗*p* < 0.001 *versus* control (vehicle treated); ^##^*p* < 0.01, ^###^*p* < 0.001 *versus* LPS-activated cells. A_2A_R, adenosine A_2A_ receptor; CtsD, cathepsin D; IPMΦ, mouse peritoneal macrophage; LPS, lipopolysaccharide; WB, Western blot.

Subsequently, we aimed to assess the effect of A_2A_R activation on the maturation of CtsD in macrophages. Thus, RAW 264.7 and IPMΦ cells were stimulated with LPS, A_2A_R agonist (CGS21680), or a combination of LPS and different concentrations of CGS21680 for 4 h, and the CtsD expression was determined by immunoblot experiments. Interestingly, while all the treatments failed to alter total CtsD density, LPS/CGS21680 treatment significantly increased the relative expression of CtsD HC in a concentration-dependent manner in RAW 264.7 and IPMΦ cells (1.25–1.50-fold and 1.20–1.90-fold, respectively) (Fig. 6). However, LPS or CGS21680 administration alone failed to change the relative expression level of CtsD HC in either cell type (Fig. 6, *A* and *B*). Next, we measured the aspartyl protease enzyme activity in macrophages using a specific fluorogenic 11-mer peptide substrate in IPMΦ cell lysates and culture media. Interestingly, the enzymatic activity significantly increased after LPS/CGS21680 treatment (Fig. 6, *C* and *D*). Thus, our results demonstrate that A_2A_R activation prompts CtsD maturation and enzymatic activity.

### A_2A_R knockout (A_2A_R^−/−^) mice show decreased CtsD protein expression in IPMΦ cells

**Figure 7:**
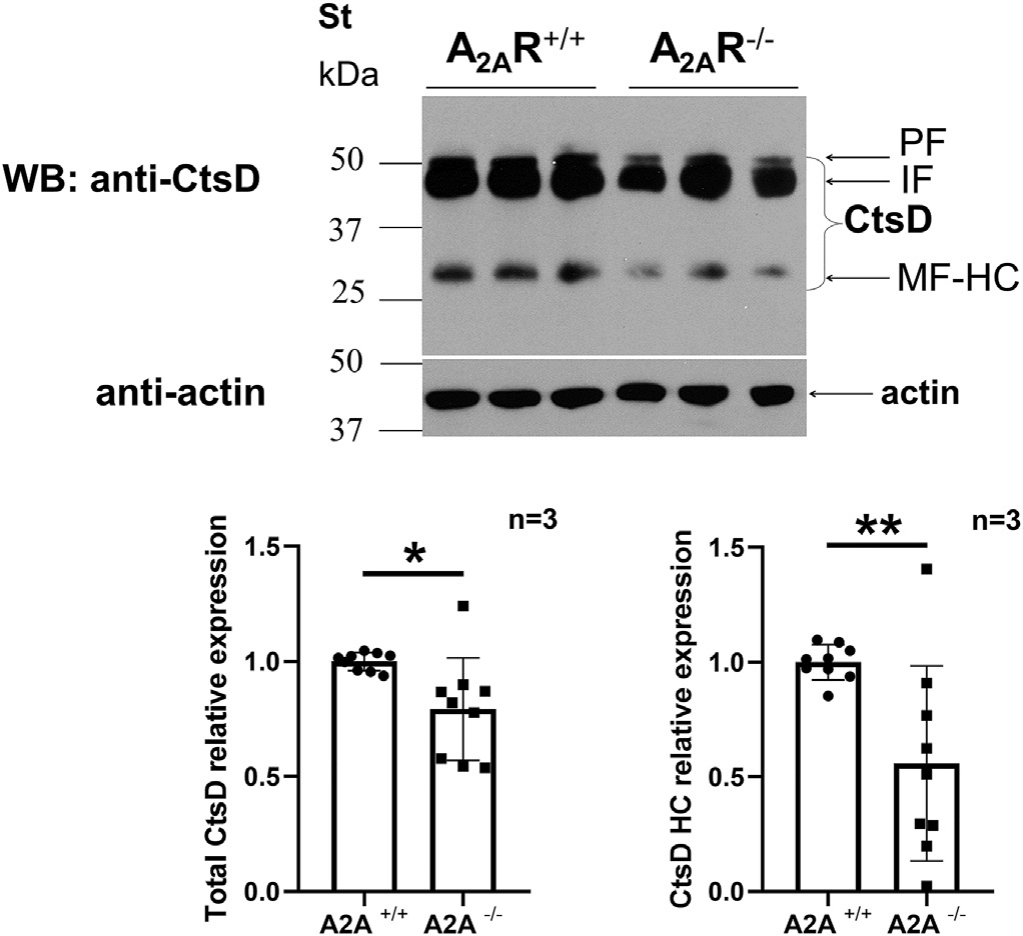
**A**_**2A**_**R**^**−/−**^**mice have decreased CtsD protein expression in IPMΦ cells.** Levels of different CtsD forms (PF, IF, and HC) and β-actin were measured in protein extracts from WT and A_2A_R^−/−^ mice in IPMΦ cells, using a CtsD-specific polyclonal antibody. Sample loading was normalized to β-actin. Statistical analyses of the relative expression of CtsD total proteins and HC form were based on three independent samples evaluated by independent *t* test. Data are presented as mean ± SD. ∗*p* < 0.0145, ∗∗*p* < 0.0074 *versus* control (A_2A_R^+/+^) cells. A_2A_R, adenosine A_2A_ receptor; CtsD, cathepsin D; HC, heavy chain; IF, intermediate form; IPMΦ, mouse peritoneal macrophage cell; PF, proform.

To further investigate the role of A_2A_R in the regulation of CtsD maturation, we examined the protein density of the different forms of CtsD in A_2A_R knockout (A_2A_R^−/−^) mice and their wildtype (A_2A_R^+/+^) littermates by immunoblot. Both the total CtsD and the active HC form (34 kDa) were present in significantly lower levels in A_2A_R^−/−^ animals when compared with A_2A_R^+/+^ mice (Fig. 7). Thus, deletion of A_2A_R results in significantly decreased CtsD HC expression.

### A_2A_R activation alters the localization of CtsD in LPS-stimulated macrophages

**Figure 8:**
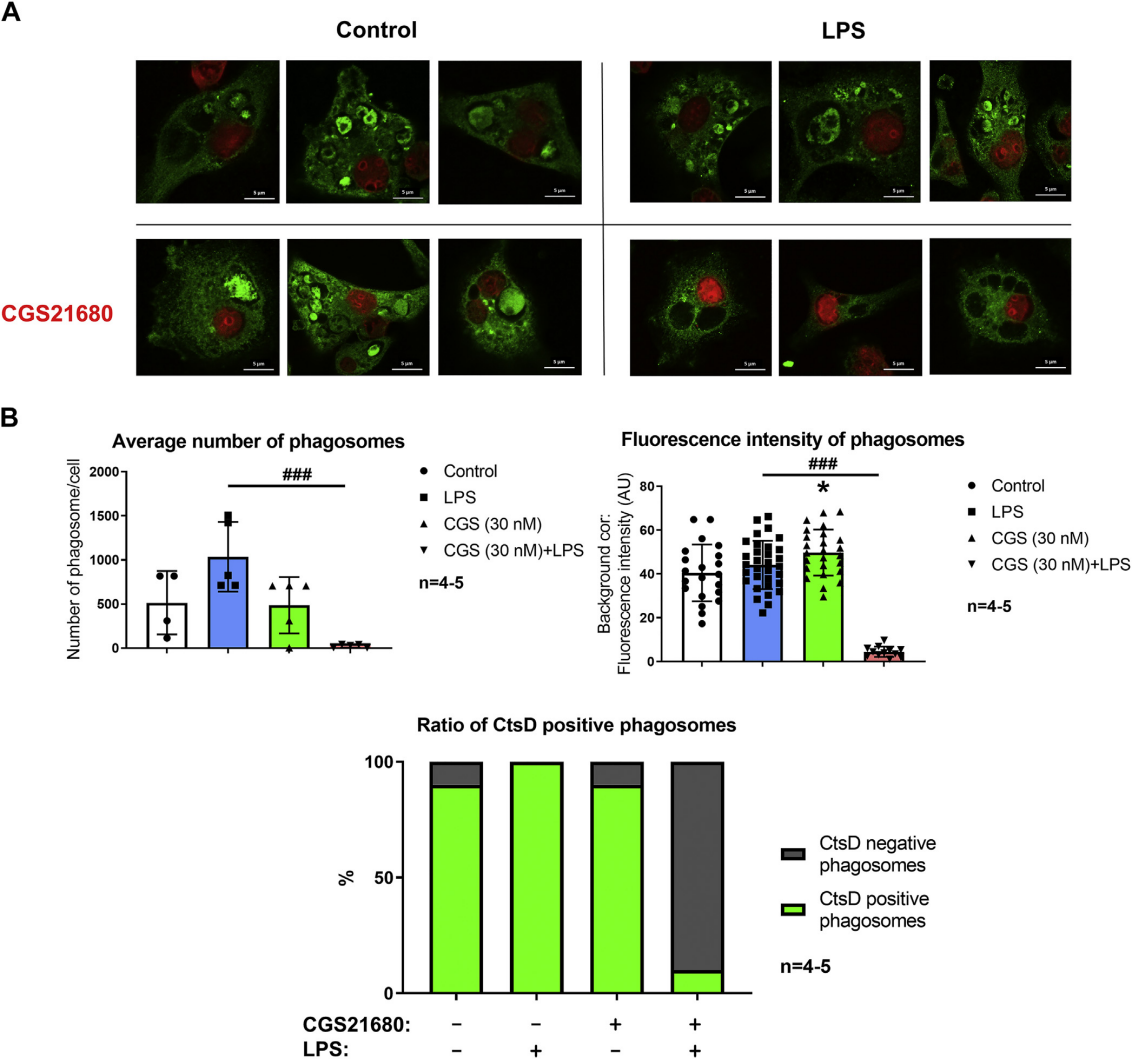
**A**_**2A**_**R activation modifies the localization of CtsD in LPS-activated macrophages.***A*, mouse IPMΦs were treated with CGS21680 (30 nM) in the absence or the presence of LPS (100 ng/ml) for 4 h. IS of mouse IPMΦs was conducted to determine the localization of endogenous CtsD protein (*green*) using CtsD-specific primary antibody and Alexa-488–conjugated secondary antibody. Nuclei of macrophages were stained with TO-PRO-3 iodide (*red*). Representative pictures are shown from 4 to 5 independent experiments. *B*, quantification of different parameters of the phagosomes from the immunostained IPMΦs was done by LAS X software (Leica Microsystem GmbH). The analysis was based on the individual densitometry of 50 different cells from 4 to 5 independent experiments. Data are presented as mean ± SD. Values from ANOVA: *F* = 52.44 and *p* < 0.001 for fluorescence intensity of and *F* = 9.022 and *p* = 0.0012 for average number of phagosomes. ∗*p* < 0.05 *versus* control (vehicle treated); ^###^*p* < 0.001 *versus* LPS-activated cells. A_2A_R, adenosine A_2A_ receptor; CtsD, cathepsin D; IPMΦ, mouse peritoneal macrophage cell; IS, immunostaining; LPS, lipopolysaccharide.

Finally, we questioned if A_2A_R activity defines the subcellular distribution of CtsD in macrophages. To this end, we assessed by immunofluorescence staining the subcellular localization of CtsD after A_2A_R activation in resting and LPS-stimulated IPMΦ cells. Indeed, resting IPMΦs cells had large phagosomes within their cytosol showing high CtsD staining, both in the absence or the presence of the A_2A_R agonist CGS21680 (Fig. 8*A*). Importantly, when IPMΦ cells were activated with LPS and treated with CGS21680, the CtsD-specific fluorescence signal was completely abolished from the phagosomes (Fig. 8*A*), thus indicating an A_2A_R-dependent subcellular redistribution in activated macrophages. Indeed, the number of phagosomes in the cells was determined, and the green fluorescence intensity of the phagosome areas was recorded in CtsD immunostained images using a Leica Application Suite X (LAS X; Leica Microsystems GmbH) software. The number of phagosomes significantly decreased (0.45-fold) after pharmacological activation of A_2A_R in LPS-stimulated IPMΦs cells. Based on CtsD-specific fluorescence intensity in the phagosomes, A_2A_R activation dramatically decreased (0.102-fold) CtsD-positive staining in LPS-stimulated IPMΦs. The calculated ratio of CtsD-positive phagosomes after A_2A_R plus LPS activation was 10% compared with the LPS-stimulated (100%) IPMΦs (Fig. 8*B*). Overall, our immunofluorescence results demonstrated that A_2A_R activation not only promotes the maturation of CtsD and enhances its enzyme activity but also alters the subcellular distribution of CtsD in stimulated IPMΦ cells.

## Discussion

In the present study, we report a new A_2A_R interactor, namely CtsD, resulting from, to our knowledge, the first-time completion of a YTH system screening in an IPMΦ cDNA library using the C-terminal tail of the receptor as a bait. This A_2A_R–CtsD interaction was first validated *in vitro* and in cellular models (*i.e.*, RAW 264.7 and IPMΦ). Then, we determined the functional consequences of this protein–protein interaction, thus revealing that A_2A_R is a substrate for CtsD and that CtsD maturation and enzymatic activity depends on A_2A_R activation in macrophages.

Cathepsins are a large family of cytosolic proteases, which have been related to inflammatory processes and to the regulation of the immunity response. Particularly, CtsD is a lysosomal aspartyl protease whose activation is associated with macrophage infiltration in adipose tissue (39). Despite the existence of some evidence indicating a relationship between cathepsins in general, and CtsD in particular, with macrophage biology, a macroperspective on this relationship is still missing. Indeed, the A_2A_R–CtsD interaction reported here might shed light on this connection.

Adenosine, through activation of A_2A_R, plays a key role in macrophage activation, thus triggering anti-inflammatory responses (1, 36, 37, 40, 41, 42) and preventing ischemia–reperfusion injury in liver (43), lung (44), and kidney (45), and in progressive kidney fibrosis (46). Thus, it seems reasonable to think that understanding the A_2A_R interactome in macrophages would be essential to understand some of the mechanisms by which A_2A_R drives adenosine-dependent macrophage function in inflammation. Macrophages secrete cytokines, free radicals, prostanoids, proteinases, and angiogenic factors to alarm the tissue of the presence of infective and injurious agents, thus orchestrating the inflammatory response toward the restoration of tissue homeostasis (reviewed in Ref. (47)). In addition, macrophages play a key role in the normal cellular turnover of organs based on their ability to phagocytose apoptotic cells, cellular debris, and matrix (reviewed in Ref. (48)). Here, we demonstrated that A_2A_R agonist activation favors the maturation and increases the enzymatic activity of CtsD in macrophages, thus boosting its cytosol and lysosomes with catalytic CtsD. Accordingly, the activation of A_2A_R in macrophages would, in theory, constitute a physiological response toward the restoration of tissue homeostasis both in health and disease (49). Therefore, it can be hypothesized that A_2A_R, by controlling CtsD activity levels in macrophages, could be behind the putative adenosine regulation of macrophage function.

GPCRs provide exquisite cellular responses to changes in the external milieu. Given their central role in mediating cellular responses, their function is tightly regulated. Upon activation, receptor signaling is limited by desensitization, internalization, recycling, and degradation events. While some of these events are well characterized for A_2A_R (50), others still are not known in full, as for many other GPCRs. Our results demonstrating the physical and functional interaction of A_2A_R with CtsD in macrophages provide a new piece of information how A_2A_R expression is regulated on macrophages. Indeed, its long C-terminal tail has been largely studied, and its impact on the function of the receptor is fairly well described. Accordingly, upon agonist binding, A_2A_R is phosphorylated at specific threonine residues located at the C-terminal domain by G protein–coupled receptor kinases (GRKs) (51), thus facilitating the binding to β-arrestin and its uncoupling from G proteins (52, 53). Subsequently, A_2A_R bound to GRK/β-arrestin internalizes into endosomes (14), from where it can take two possible trafficking routes: (i) recycled back to the plasma membrane upon dephosphorylation and GRK–arrestin dissociation (54) and (ii) ubiquitination and lysosomal degradation (20, 55). Importantly, we found that A_2A_R undergoes proteolysis by CtsD, likely in lysosomes, thus limiting the expression of the receptor in macrophages. Of note, although other A_2A_R interactors regulate receptor’s expression, internalization, and trafficking (56), none of these take part in its degradation, indicating a unique function for CtsD in A_2A_R physiology. Thus, the A_2A_R can be added to the list of GPCRs whose degradation by lysosomal enzymes occurs, and which include β_2_-adrenoceptors, (57) C-X-C chemokine receptor type 4 (58) and protease-activated receptor 2 (59), among others.

The role of lysosomal proteases in the immune function has been studied extensively (60), not only limited to their active participation in antigen presentation and/or the trafficking of growth factor receptors (61) but also with regard to their catalytic role at the extracellular milieu upon release. However, the functional consequences of their interaction with specific GPCRs in macrophages have not been reported yet. Accordingly, here we describe for the first time the interaction of a lysosomal protease (*i.e.*, CtsD) with a GPCR (*i.e.*, A_2A_R) in macrophages, thus adding a new partner to the list of A_2A_R-interacting proteins (13, 19, 23); for review, see Ref. (56), the first in an immune cell.

In conclusion, we found that A_2A_R is proteolytically degraded by CtsD and that A_2A_R agonist activation favors the maturation and increases the enzymatic activity of CtsD in macrophages. Thus, our data suggest that this A_2A_R–CtsD interaction in macrophages may represent an autologous control strategy for tuning adenosine signaling in inflammatory processes.

## Experimental procedures

### Materials

All materials were ordered from Sigma–Aldrich, except for cDNA (EX-Mm01091-Lv113; Genecopoeia); NdeI, SalI, SfiI, SpeI, and XhoI restriction enzymes (New England Biolabs); Falcon Multiwell tissue culture plates (catalog no.: 351146; BD Biosciences); RAW 264.7 and HEK-293 cells (American Type Culture Collection); T75 cell culture dishes (catalog no.: Z707546; TPP); Bicinchoninic acid (BCA) Protein Assay Kit (catalog no.: 23225), West Pico Super Signal ECL Western Blotting (WB) Detection Reagent (catalog no.: 34580), Dynabeads Protein G magnetic beads suspension (catalog no.: 10003D), Coomassie brilliant blue G-250 dye (catalog no.: 20279), and Protein MagicMarker standard (catalog no.: LC5602) (Thermo Fisher Scientific); SDS-PAGE (catalog no.: 4568034), Precision Plus Protein Dual Color Standards (catalog no.: 1610374) (Bio-Rad); ELISA DuoSet kits (catalog nos.: DY417 and DY406), CtsD enzyme (catalog no.: 1029-AS), immunoglobulin G (IgG) isotype control (catalog no.: AB-108-C) (R&D Systems); Calbiochem fluorogenic 11-mer peptide substrate (catalog no.: 219360) and PMSF-RO (Merck); CD11b-MACS cell separation column (catalog no.: 130-049-601) (Miltenyi Biotec); NucleoTrap mRNA kits (catalog no.: 740656), ChromaSpin purification column (catalog no.: 740955) (Macherey-Nagel); Advantage 2 PCR Enzyme System with trypsin inhibitor gene from calf thymus DNA (catalog no.: 639202), SMART cDNA Library Construction Kit with mouse liver double-stranded cDNA control library and linearized pGADT7-Rec “prey” vector (catalog no.: 634901), Matchmaker Gold Yeast Two-Hybrid System (catalog no.: 630489), plasmid cytomegalovirus (pCMV)-cMyc vector (catalog no.: 635689) (Takara-Clontech); pET42a vector (catalog no.: 70561), *E. coli* BLR strain (catalog no.: 69053; Merck-Novagen); jetPRIME transfection reagent (catalog no.: 114-15; Polyplus transfection); GST-specific MagneGST Protein Purification System (catalog no.: V8600; Promega); 8-well tissue culture–treated glass chamber slides (catalog no.: 354118; Falcon); 96-well Cell Carrier Ultra plate (catalog no.: 6055302; PerkinElmer); CGS21680 (catalog no.: 1063; Tocris); and Protease Inhibitor Cocktail (PIC; catalog no.: M221; VWR International). Antibodies used for WB, IP, immunostaining (IS), and nuclear staining dyes used for IS are summarized in Table S1.

### Plasmid constructs

The C-terminal 855 to 1233 nucleotides of the Adora2a coding sequence (CDS) flanked by NdeI and SalI ends were amplified using the full-length cDNA (EX-Mm01091-Lv113; Genecopoeia) as a template. The PCR fragment was inserted into the “bait” pGBKT7 vector previously linearized by NdeI and SalI restriction endonucleases. The A_2A_R C-terminal sequence was cloned in frame with the GAL4 DNA-BD and cMyc epitope tag through the restriction sites engineered into the PCR primers. After the transformation of the construct into the yeast Y2H Gold strain, the cells expressed the C-terminal 126 amino acid residues of A_2A_R (A_2A_R^284–410^) with GAL4 DNA-BD as a fusion protein. To express GST-A_2A_R^284–410^ recombinant protein in *E. coli*, we cloned the CDS (855–1233 nucleotides) flanked by SpeI and XhoI ends into the pET42a vector SpeI and XhoI restriction sites. The A_2A_R C-terminal sequence was cloned in frame with GST protein. After transformation of the construct into the *E. coli* BLR strain, the cells expressed GST-A_2A_R^284–410^ as a fusion protein. For mammalian cell expression of cMyc-A_2A_R^284–410^ recombinant protein, we used the pCMV vector and cloned the CDS of A_2A_R^284–410^ (855–1233 nucleotides) into the SfiI and XhoI restriction sites of the vector. Oligos used for cloning the 855 to 1233 nucleotides of the A_2A_R CDS are summarized in Table S2.

### Isolation of IPMΦs

Mice (C57BL6/J wildtype [A_2A_R^+/+^] and A_2A_R-knockout [A_2A_R^−/−^]) were injected intraperitoneally with 2 ml of sterile Brewer's thioglycolate broth (4% w/v). Four days later, the mice were terminated by cervical dislocation, and peritoneal macrophage cells (IPMΦ) were harvested using 10 ml of Dulbecco's modified Eagle's medium (DMEM; catalog no.: LM-D1111; GENTAUR Europe BVBA). The cells were centrifuged for 10 min at 300*g* at 4 °C. The cells were suspended in 2 ml red blood cell lysis buffer (155 mM NH_4_Cl, 10 mM KHCO_3_, and 0.1 mM EDTA), incubated for a few minutes on ice, and the volume was brought up to 10 ml with DMEM solution. Macrophages were centrifuged again for 10 min at 300*g* at 4 °C and resuspended in DMEM containing 10% fetal bovine serum (FBS), 50 U/ml penicillin, 50 μg/ml streptomycin, and 1.5 mg/ml sodium bicarbonate. Cells were seeded into Falcon Multiwell tissue culture plates. The dishes were then incubated at 37 °C in a humidified incubator for 5 h to allow the cells to adhere to the surface of the dishes. Nonadherent cells were removed by washing with serum-free DMEM, and the cells were suspended again in DMEM containing 10% FBS. About 18 h after the initial plating, the macrophages were treated with the various pharmacological compounds.

### Cell culture

RAW 264.7 macrophages, primary IPMΦ cells, and HEK-293 cells permanently expressing the human A_2A_R tagged with FLAG and SNAP protein at its N terminus (*i.e.*, HEK-293-FLAG-A2AR^SNAP^ cells) (62) were cultured in high-glucose DMEM (catalog no.: LM-D1111; GENTAUR Europe BVBA) containing 10% FBS (catalog no.: FB-1090; GENTAUR Europe BVBA), 50 U/ml penicillin, 50 μg/ml streptomycin (catalog no.: P4333; Sigma–Aldrich), and 2 mM l-glutamine (catalog no.: 59202C; Sigma–Aldrich) in T75 cell culture dishes. Cells were cultured in a humidified (80%) incubator at a constant temperature (37 °C) and CO_2_ concentration (5 v/v%).

### Animal models

The experiments were carried out with 8- to 12-week-old C57BL6/J wildtype (A_2A_R^+/+^) mouse colonies (The Jackson Laboratory) and A_2A_R-knockout (A_2A_R^−/−^) mouse colonies that were established *via* heterozygous breeding in the laboratory of George Haskó (Department of Anesthesiology, Irving Medical Center, Columbia University, New York). All mice were maintained in specific pathogen-free conditions in the Central Animal Facility, and all animal experiments were conducted according to the guidelines of the Declaration of Helsinki and approved by the Institutional Review Board of the University of Debrecen (DEMÁB).

### Pharmacological treatment of macrophages

The pharmacological agents were dissolved in water at the following concentrations: Pepstatin A penetratin, 1.5 mM; CGS21680, 50 mM; and LPS (catalog no.: L-3880; Sigma–Aldrich), 5 mg/ml. Pepstatin A penetratin was used at a final concentration of 3 or 9 μM. The final concentration of CGS21680 was 100 nM, and LPS was utilized at a concentration of 100 ng/ml in the culture media. We preincubated the macrophages in the presence of Pepstatin A penetratin or CGS21680 for 20 min before LPS was added for 4 h to the media. After the treatment, the cells were either immunostained and analyzed by confocal microscopy or lysed, and protein concentration was determined from the cell lysate. Cytokine levels were measured from the media, and aspartic protease activity was measured from the cell lysate.

### Protein isolation

After treatment with the different drugs, the RAW 264.7 and IPMΦ cells were washed with PBS solution and then lysed in ice-cold modified radioimmunoprecipitation assay (RIPA) buffer (50 mM Tris–HCl, 150 mM NaCl, 1 mM EDTA, 0.25% sodium deoxycholate, 1% NP-40, 1× PIC [catalog no.: M221; VWR International], and 1 mM PMSF). The lysate was then centrifuged (10,000*g* for 10 min at 4 °C), and the supernatants were collected. Protein concentration was determined by Direct Detect Spectrophotometer (Merck-Millipore) and BCA Protein Assay Kit (catalog no.: 23225; Thermo Fisher Scientific). A standard curve was prepared by plotting the absorbance value at a wavelength of 562 nm corrected to the value of the blank sample for each bovine serum albumin (BSA) standard. Then, the standard curve was used to determine the protein concentration of each sample.

### Immunoblot

Ten micrograms of protein lysates from each sample were denatured for 10 min at 95 °C in the presence of SDS sample buffer and separated on a 4 to 12% gradient gel (NuPAGE; Thermo Fisher Scientific) or 10% SDS-PAGE (Bio-Rad Laboratories) at 100 V for 60 min. The separated proteins were transferred to a nitrocellulose membrane (catalog no.: 10600016; Amersham; Sigma–Aldrich) at 400 mA for 90 min. After blocking with 3% BSA in 1× Tris-buffered saline with Tween-20 buffer, membranes were incubated with cMyc-specific (Sigma–Aldrich), GST-specific (Ilona Farkas), CtsD-specific (catalog no.: AF1029; R&D Systems), or FLAG-specific antibody (catalog no.: mab90006-P; Covalab) overnight at 4 °C. The following day, membranes were incubated with antigoat-horse radish peroxidase (HRP) (catalog no.: A15999; Novex by Life Technologies), antimouse-HRP (catalog no.: 7076S; Cell Signaling), and β-actin (catalog no.: 47778 HRP; Santa Cruz) antibodies for 1 h at room temperature. Bands were detected using the ECL WB Detection Reagent (catalog no.: 34580; Super Signal, West Pico; Thermo Fisher Scientific). The signal was detected with Chemidoc Touch Imaging System (Bio-Rad Laboratories) and quantified by Image Lab (Bio-Rad Laboratories) and ImageJ software (National Institutes of Health).

### ELISA

Cytokine (IL-10 and IL-6) concentrations from the cell culture media were determined using commercially available ELISA DuoSet kits (catalog nos.: DY417 and DY406; R&D Systems) according to the manufacturer’s instructions.

### Aspartic protease activity measurement

Aspartic protease activity from the cell lysate (protease inhibitors were not added to the lysis buffer) and from the cell culture media was measured by the hydrolysis of the fluorogenic 11-mer peptide substrate (7-methoxycoumarin-4-yl)acetyl-Gly-Lys-Pro-Ile-Leu-Phe-Phe-Arg-Leu-Lys(Dnp)-D-Arg-NH_2_, (catalog no.: 219360; Merck-Calbiochem). Enhanced fluorescence after the cleavage of the Phe-Phe amide bond was spectrofluorimetrically determined at a pH of 4. Reaction mixtures contained 40 μl of 50 mM sodium acetate buffer, pH 4.0; 5 μl of 200 μM substrate solution; and 5 μl of sample (cell lysate or macrophage culture media). The reaction mixtures were incubated at 40 °C for 10 min, and the reactions were terminated with 200 μl of 10% trichloroacetic acid. The increase in fluorescence intensity produced by substrate cleavage during incubation was measured at an emission wavelength of 393 nm with excitation at 328 nm using a BioTek Synergy2 fluorescence spectrophotometer (Agilent Technologies). The specific enzyme activity was normalized to the protein concentration of the cell lysate or the cell culture media.

### CtsD protease treatment of HEK-293-FLAG-A_2A_R^SNAP^ cell lysates and GST-A_2A_R^284–410^, GST recombinant proteins

After washing HEK-293-FLAG-A_2A_R^SNAP^ cells once with ice-cold PBS, the cells were lysed in ice-cold RIPA buffer (50 mM Tris–HCl [pH 7.4], 1% NP-40, 0.5% sodium deoxycholate, 0.1% SDS, 150 mM NaCl, 2 mM EDTA, and 50 mM NaF). Then, cells were centrifuged at 10,000*g* for 10 min at 4 °C. The supernatants were collected, and protein concentrations were determined by Direct Detect Spectrophotometer (Merck-Millipore). Cell lysates (50 μg protein) were incubated with recombinant mouse CtsD (catalog no.: 1029-AS; R&D Systems) at a concentration of 1.5 μg/ml for 1 and 2 h at 37 °C. GST-A_2A_R^284–410^ and GST recombinant proteins (200 ng) were incubated with recombinant mouse CtsD at a concentration of 10 μg/ml for 5, 10, and 30 min, at 37 °C. Before this procedure, the CtsD was preincubated at room temperature for 10 min. The mixture was supplemented with assay buffer (0.1 M sodium acetate, 0.2 M NaCl, pH = 3.5). After the incubation of mammalian cell lysate and CtsD, ten times the sample volume of cold (−20 °C) acetone was added to the mixture, then the samples were vortexed, and incubated for 30 min at −20 °C. The mixture was centrifuged at 10,000*g* for 10 min at 4 °C. The supernatants were removed, and the pellets were dissolved in 72 μl RIPA buffer and supplemented with 18 μl 5× SDS sample buffer. After the incubation of GST-A_2A_R^284–410^, GST proteins, and CtsD in a 48 μl assay buffer, the enzyme reactions were stopped with 12 μl 5× SDS sample buffer. Then all samples were denatured for 10 min at 95 °C and separated by 10% SDS-PAGE electrophoresis. While HEK-293-FLAG-A_2A_R^SNAP^ cell samples were analyzed by WB using anti-FLAG antibody (catalog no.: mab90006-P; Covalab), GST-A_2A_R^284–410^, GST protein samples were visualized by silver staining in the gel.

### Mice peritoneal macrophage cDNA library construction

For the cDNA library construction, we used 2 μg of total RNA isolated from mouse (C57BL6/J) macrophages that were harvested as described previously and purified using a CD11b-MACS cell separation column (catalog no.: 130-049-601; Miltenyi Biotec). The purification resulted in 98.5% IPMΦ homogeneity, as determined by flow cytometry with CD11b-specific and F4/80-specific antibody labeling. The poly(A) RNA was purified and enriched using a NucleoTrap mRNA kit (catalog no.: 740656; Macherey-Nagel). Purified poly(A)-RNA (0.05 μg) was reverse transcribed, and the cDNA was amplified in a long PCR according to the user manual of the Advantage 2 PCR Enzyme System (catalog no.: 639202; Takara-Clontech). The double-stranded PCR product was further purified and enriched using ChromaSpin purification column (catalog no.: 740955; Macherey-Nagel) and used for cDNA library construction according to the SMART cDNA Library Construction Kit (catalog no.: 634901; Takara-Clontech) (Fig. S1*A*). The quality of the mouse IPMΦ cDNA library was tested by PCR amplification of six different genes (AdoraA1, Adora2a, and Adora2b, F4/80 receptor, TNFα, and PPARγ) and then compared with the PCR products of the same genes using mouse liver double-stranded cDNA control library as a template (including in SMART cDNA Library Construction Kit) (Fig. S1*B*). Then, 2.3 μg double-stranded cDNA library was cotransformed with 4 μg linearized pGADT7-Rec “prey” vector into the *S. cerevisiae* Y187 strain. All yeast growing media were supplemented with kanamycin (50 μg/ml). The efficiency of the recombination was tested on synthetic dropout plates lacking leucine and tryptophan (SD/-Leu/-Trp). Transformed yeast cells were plated on SD/-Leu/-Trp selective media in 100× dilution and incubated for 3 days at 30 °C. Nontransformed Y187 yeast cells were used as a negative control, and 25 ng SV40 large T PCR product with 0.5 μg pGADT7-Rec vector (catalog no.: 634901; Takara-Clontech) were cotransformed into Y187 yeast cells as a positive control. The library scale transformation resulted in 4.9 × 10^9^ independent yeast colonies, which were able to grow on selective media (Fig. S1*C*). All colonies were collected and stored in small aliquots for further mating process in the YTH screen.

### Y2H screening and retransformation

The Y2H screen was carried out according to the user manual of Matchmaker Gold Yeast Two-Hybrid System (catalog no.: 630489; Takara-Clontech). The pGADT7-cDNA library “prey” vector and the pGBKT7 “bait” vector (catalog no.: 630489; Takara-Clontech) were transformed into the Y187 and Y2H Gold yeast strains, respectively. Y2H Gold strains harboring pGBKT7 plasmids were maintained in SD/-Trp media, whereas Y187 strains harboring pGADT7 plasmids were maintained in SD/-Leu media. Prior to mating, the individual Y2H Gold strain containing the sequences of BD-cMyc-A_2A_R^284–410^ was plated on SD/-Ade/-His/-Trp agar supplemented with 40 mg/ml X-alpha-Gal and determined not to be an autoactivator of the reporter genes. The expression of GAL4 DNA-BD fusion proteins (BD-cMyc-A_2A_R^284–410^; BD-cMyc-p53; BD-cMyc-Lamin) was tested by WB using cMyc-specific antibody (Fig. S2*A*). The two haploid strains were cultured together to mate and create diploid yeast cells. Diploid cells contained four reporter genes (*AUR1-C*, *ADE2*, *HIS3*, and *MEL1*) that were activated only in response to two-hybrid interactions. After mating, the diploid cells were plated on the double dropout media (SD/-Leu/-Trp supplemented with 40 μg/ml X-alpha-Gal and 125 ng/ml Aureobasidin A). The blue yeast colonies were picked and plated onto the quadruple dropout media (SD/-Ade/-His/-Leu/-Trp supplemented with 40 μg/ml X-alpha-Gal and 125 ng/ml Aureobasidin A). We identified 23 positive clones from 4.6 × 10^9^ screened yeast colonies (Fig. S2*B*) with “bait” and “prey” plasmids and express proteins that interact with each other to activate *HIS3* and *ADE2* genes. From the 23 positive clones, 15 encoded CtsD aspartyl protease. The interaction between the Gal4 BD-cMyc-A_2A_R^284–410^ and Gal4 AD-CtsD^321–410^ was confirmed by four independent yeast retransformations using the cDNA-containing plasmids. The pGBKT7-p53 and pGADT7-SV40 small T antigen as well as pGBKT7-p53 and pGADT7-Lamin cotransformations were used as positive and negative controls, respectively.

### Expression of cMyc-A_2A_R^284–410^ recombinant protein in RAW 264.7 cells

The coding regions of A_2A_R^284–410^ were cloned into the multiple cloning site of pCMV-cMyc vector (catalog no.: 635689; Takara-Clontech) producing fusion proteins with an N-terminal cMyc peptide tag. RAW 264.7 macrophage cells were transfected with pCMV-cMyc-A_2A_R^284–410^ vector using jetPRIME transfection reagent (catalog no.: 114-15; Polyplus-transfection) according to the manufacturer’s instructions. The optimal plasmid DNA (in microgram):transfection reagent (in microliter) ratio was 1:3. Cells were cultured in a humidified (80%) incubator at constant temperature (37 °C) and CO_2_ concentration (5 v/v%). The expression of cMyc-A_2A_R^284–410^ recombinant protein was detected by WB using a cMyc-specific antibody (Sigma–Aldrich).

### IP of cMyc-A_2A_R^284–410^, full length A_2A_R, and CtsD proteins

RAW 264.7 cell lysates containing 500 μg of protein extract in RIPA buffer were diluted with PBS and supplemented with 100× PIC (catalog no.: M221; VWR International) and 100× PMSF to 500 μl. About 10 μg anti-CtsD antibody and its isotype control IgG (catalog nos.: AF1029 and AB-108-C; R&D Systems) or 1 μg anti-cMyc antibody and its isotype control IgG_1_ (catalog nos.: M5546 and I5006; Sigma–Aldrich) or 8.5 μg anti-A_2A_R (catalog no.: AAR-002; Alomone Labs) were added to the complex and incubated overnight with rotation at 4 °C. On the following day, equilibrated magnetic bead suspension (catalog no.: 10003D; Dynabeads Protein G; Thermo Fisher Scientific) was added to the lysates and incubated for 1 h with rotation at 4 °C. The beads were washed three times with RIPA–PBS buffer (1:2 ratio) supplemented with 100× PIC and 100× PMSF. Bound proteins were eluted from the immune complexes with 48 μl ice-cold RIPA buffer containing 12 μl 5× SDS sample buffer. The eluted samples were denatured for 10 min at 95 °C, separated by 10% SDS-PAGE electrophoresis, and analyzed by WB using anti-cMyc antibody and anti-CtsD antibodies.

### Endogenous CtsD protein PD with GST-A_2A_R^284–410^

The GST and GST-A_2A_R^284–410^ recombinant proteins were expressed in the *E. coli* BLR strain and extracted from the supernatant of bacterial cells using the GST-specific MagneGST Protein Purification System (catalog no.: V8600; Promega). One-tenth of a 100 ml bacterial culture was suspended in 1 ml of lysis buffer (10 mM Tris buffer [pH 8], 150 mM NaCl, and 1 mM EDTA) supplemented with 10 μl PIC 100×, 10 μl PMSF 100×, and 10 μl lysozyme (100 μg/ml), and vortexed vigorously. The mixtures were incubated on ice for 15 min, completed with 50 μl DTT (100 mM) and 75 μl Sarkosyl (20 v/v%), vortexed again, and sonicated three times (50 cycles, five microtip limits) for 30 s with short breaks (Branson Sonifer 250). The lysates were centrifuged at 10,000*g* for 5 min at 4 °C, and 11.5 μl Triton-X solution (10 v/v%) and 30 μl pre-equilibrated magnetic beads were added to the supernatants. The mixtures were incubated overnight at 4 °C with continuous shaking, and the next day, the GST-specific complexes were washed twice with 250 μl washing buffer and twice with 250 μl PBS. Then, one-fifth of the washed GST, GST-A_2A_R^284–410^-bound beads, was eluted in 33 μl elution buffer (50 mM reduced glutathione, 50 mM Tris buffer [pH 8.1]) for 15 min at 4 °C with continuous shaking. The mixture was denatured for 10 min at 95 °C in the presence of SDS sample buffer and separated on 10% SDS-PAGE gel at 100 V for 60 min. The separated proteins were visualized by Coomassie brilliant blue G250 staining, and the signal was detected with Fluorochem FC2 Imaging System (Alpha Innotech). For CtsD PD experiments, 145 μg of total protein containing IPMΦ cell lysates were incubated with two-fifths of the washed GST or GST-A_2A_R^284–410^-bound beads in 500 μl final volume of RIPA buffer supplemented with 1× PIC for 2 h at 4 °C. Then, the PD complexes were washed once with 600 μl of RIPA buffer and twice with PBS supplemented with 1× PIC. Bound proteins were eluted from the PD complexes with 100 μl of ice-cold RIPA buffer containing 33 μl 3× SDS sample buffer. The eluted samples were denatured for 10 min at 95 °C, separated by gradient (4–12%) SDS-PAGE electrophoresis, and analyzed by WB using a CtsD-specific antibody (catalog no.: MAB1029; R&D Systems).

### IS of A_2A_R and CtsD protease

IPMΦ cells (10^5^) were cultured in 200 μl DMEM in 8-well tissue culture plates with treated glass chamber slides (Falcon) or 96-well Cell Carrier Ultra plates (catalog no.: 6055302; PerkinElmer). Cells were treated before IP with Pepstatin A penetratin (1, 3, and 9 μM; catalog no.: 516483; Sigma–Aldrich) or A_2A_R agonist (catalog no.: CGS21680; 100 nM) for 20 min before LPS (100 ng/ml) was added for 4 h to the cells. After the treatment, the cell culture media were changed to fresh DMEM. The A_2A_R was detected by live-cell staining using an anti-A_2A_R antibody (catalog no.: AAR-002; Alomone Labs) at a concentration of 2 μg/ml. Cells were further incubated for 30 min at 37 °C. Then, cells were fixed with 4% w/v paraformaldehyde solution for 20 min and incubated in blocking buffer (2 w/v% of BSA dissolved in PBS) at room temperature for 30 min. In CtsD-specific IS, the cells were fixed with 4% w/v paraformaldehyde solution for 20 min and incubated in blocking buffer (2 w/v% of BSA dissolved in PBS) at room temperature for 30 min. Anti-CtsD antibody (catalog no.: AF1029 or MAB1029; R&D Systems) was added (1 μg/ml or 2.5 μg/ml, respectively), and cells were incubated overnight at 4 °C. Cells were washed three times with 300 μl PBS and then Alexa-488–conjugated anti-rabbit (catalog no.: A27034; Thermo Fisher Scientific) for A_2A_R staining; Alexa-488–conjugated antirat (catalog no.: A21469; Thermo Fisher Scientific) for CtsD (catalog no.: MAB1029; R&D Systems) staining; or Alexa-647–conjugated antigoat secondary antibody (catalog no.: A27018; Thermo Fisher Scientific) were added in a concentration of 5 μg/ml to the blocking buffer and incubated for 1 h at room temperature. Nuclei were stained with 20 μg/ml 4′,6-diamidino-2-phenylindole (DAPI) (catalog no.: D1306; Thermo Fisher Scientific) or 1 μM TO-PRO-3 (catalog no.: T3605; Thermo Fisher Scientific) for 1 h at room temperature in the blocking buffer. After staining, chamber slides were covered with 5 μl of Mowiol-DABCO mounting medium (catalog no.: 81381; Sigma–Aldrich) and coverslips. Photos were taken with a Leica SP8 confocal microscope using a 63× oil immersion objective (numerical aperture [NA], 1.4). Densitometry analysis was completed by Leica LAS X Life Science software version. Fifty cells were selected per treatment in every experiment, and the cell outline was designated. Then, the Pearson coefficient was determined. The statistical analyses were made using Excel 2013. In the 96-well Cell Carrier Ultra plate (catalog no.: 6055302; PerkinElmer), the blocking buffer was changed to 50 μl PBS, and images were acquired on an Opera Phenix High Content Confocal System (PerkinElmer). A total of 190 fields and 2000 to 3000 cells were acquired per well, and laser-based autofocus was performed for each imaging position. Images of DAPI and Alexa-488 channels were collected at 2 μm of Z image plane using a 63× water immersion objective (NA, 1.15) to visualize the cells and the localization of A_2A_R. The primary data were analyzed using Harmony 4.8 software (PerkinElmer) according to the Spot Analyses Ready to Made Solution (http://www.perkinelmer.com/product/harmony-4-2-office-hh17000001) with custom modifications. Image intensities were rescaled, cells were identified using the DAPI signal, and the cellular phenotypes were characterized based on the Alexa-488 signal. Cellular features, such as the number of spots, total spots area, relative spot intensity in the membrane, and cytoplasm regions, were extracted. The statistical analyses of the parallel dataset were made using GraphPad Prism 7 (GraphPad Software, Inc) program. The evaluation of the data was based on the individual analysis of 2000 to 3000 different cells and presented as mean ± SEM. ∗*p* < 0.05, ∗∗*p* < 0.01, and ∗∗∗*p* < 0.001 *versus* control (vehicle treated); ^#^*p* < 0.05, ^##^*p* < 0.01, and ^###^*p* < 0.001 *versus* LPS-treated cells.

### Statistical analyses

Data are presented as mean ± SD of 3 to 5 independent experiments. D’Agostino and Pearson test were used to analyze normality. In that case, when the data show normal distribution, one-way ANOVA was performed complemented with Sidak's post hoc test. In the other case, if the data does not show normal distribution, the data were transformed than one-way ANOVA was performed complemented with Sidak’s post hoc test. In Figure 7, our data showed normal distribution; two groups of samples were compared statistically by independent *t* test. *p* Values <0.05 were considered statistically significant (∗*p* < 0.05; ∗∗*p* < 0.01; and ∗∗∗*p* < 0.001). Statistical analyses were performed with GraphPad Prism 8.0 software.

## Data availability

The cDNA library in *S. cerevisiae* Y187 strain from IPMΦs is available *via* material transfer agreement from E. Kokai. Data of Figures 1 and 2 and Figs. S1 and S2 are contained within the article. Raw data of the Figure 3, Figure 4, Figure 5, Figure 6, Figure 7, Figure 8 are available at http://193.6.152.202:5000/sharing/kMa31reJQ URL address, and the raw data of high content confocal microscope images and analysis are available upon request from the corresponding author (ekokai@med.unideb.hu).

## Supporting information

This article contains supporting information.

## Conflict of interest

The authors declare that they have no conflicts of interest with the contents of this article.
