# Supplementary material for: Cathepsin D interacts with adenosine A2A receptors in mouse macrophages to modulate cell surface localization and inflammatory signaling
Source: J Biol Chem. 2022 Mar 31;298(5):101888. doi: 10.1016/j.jbc.2022.101888 (PMC9065627; doi:10.1016/j.jbc.2022.101888)
Supplement: Supplemental Figures S1–S3 and Tables S1, S2 [file mmc1.docx]

**Supporting Information**

Table S1. Antibodies used for Western blotting (WB), immunoprecipitation (IP), immunostaining (IS); nuclear staining dyes used for IS

| Antibody | Methods | Applied concentration | Source information |
| --- | --- | --- | --- |
| anti-cMyc | WB  IP  IS | 1.67 µg/ml  16.5 µg/ml  33.4 µg/ml | M5546 Sigma-Aldrich Kft., (Budapest, Hungary) |
| anti-CtsD | WB  IP  IS | 0.2 µg/ml  20 µg/ml  4 µg/ml | AF1029; R&D Systems (Minneapolis, MN, USA) |
| anti-CtsD | WB | 1 µg/ml | MAB1029; R&D Systems (Minneapolis, MN, USA) |
| anti- A_2A_R | WB  IS | 0.7 µg/ml  14 µg/ml | AAR-002; Alomone labs  (Jerusalem, Israel) |
| anti-Flag | WB | 0.5 µg/ml | mab90006-P; Covalab  (California, CA, USA) |
| anti-β-Actin | WB | 0.02 µg/ml | sc-47778 HRP; Santa Cruz Biotechnology (Dallas, TX, USA) |
| anti-GST | WB | 0.5 µg/ml | From Ilona Farkas, University of Debrecen, Department of Medical Chemistry |
| anti-Goat-HRP | WB | 0.2 µg/ml | A15999; Thermo Fisher  (Waltham, MA, USA) |
| anti-Mouse-HRP | WB | 0.2 µg/ml | 7076S; Cell Signaling Technology (Danvers, MA, USA) |
| anti-Rabbit-HRP | WB | 0.2 µg/ml | 7074S; Cell Signaling Technology (Danvers, MA, USA) |
| anti-Rabbit-Alexa-488 | IS | 5 µg/ml | A27034; Thermo Fisher  (Waltham, MA, USA) |
| anti-Goat-  Alexa-647 | IS | 5 µg/ml | A21469; Thermo Fisher  (Waltham, MA, USA) |
| DAPI | IS | 20 µg/ml | D1306, Thermo Fisher  (Waltham, MA, USA) |
| TO-PRO3 | IS | 1 µg/ml | T3605, Thermo Fisher  (Waltham, MA, USA) |

Table S2. Oligos used for cloning the 855-1233 nucleotides of the A_2A_R coding sequence

| Cloning vector | Forward | Reverse |
| --- | --- | --- |
| pGBKT7 | Adora2a-For-Nde I  5'-CCGA**CATATG**GCCTACAGGATCCGGGAGT-3' | Adora2a-Rev-Sal I  5’-CCA**GTCGAC**ACTCGAGCTAGGAAGGGG-3’ |
| pET42a | Adora2a-For-Spe I  5’-CCGA**ACTAGT**GCCTACAGGATCCGGGAGT-3’ | Adora2a-Rev-Xho I  5’-CCAGTCGACA**CTCGAG**GTAGGAAGGGG-3’ |
| pCMV | Adora2a-For-Sfi I  5’-GGAT**GGCCATGGAGGCC**TACAGGATCCGGGAGT | Adora2a-Rev-Xho I  5’-CCAGTCGACA**CTCGAG**GTAGGAAGGGG-3’ |

Bold letters: restriction enzyme recognition sites

**
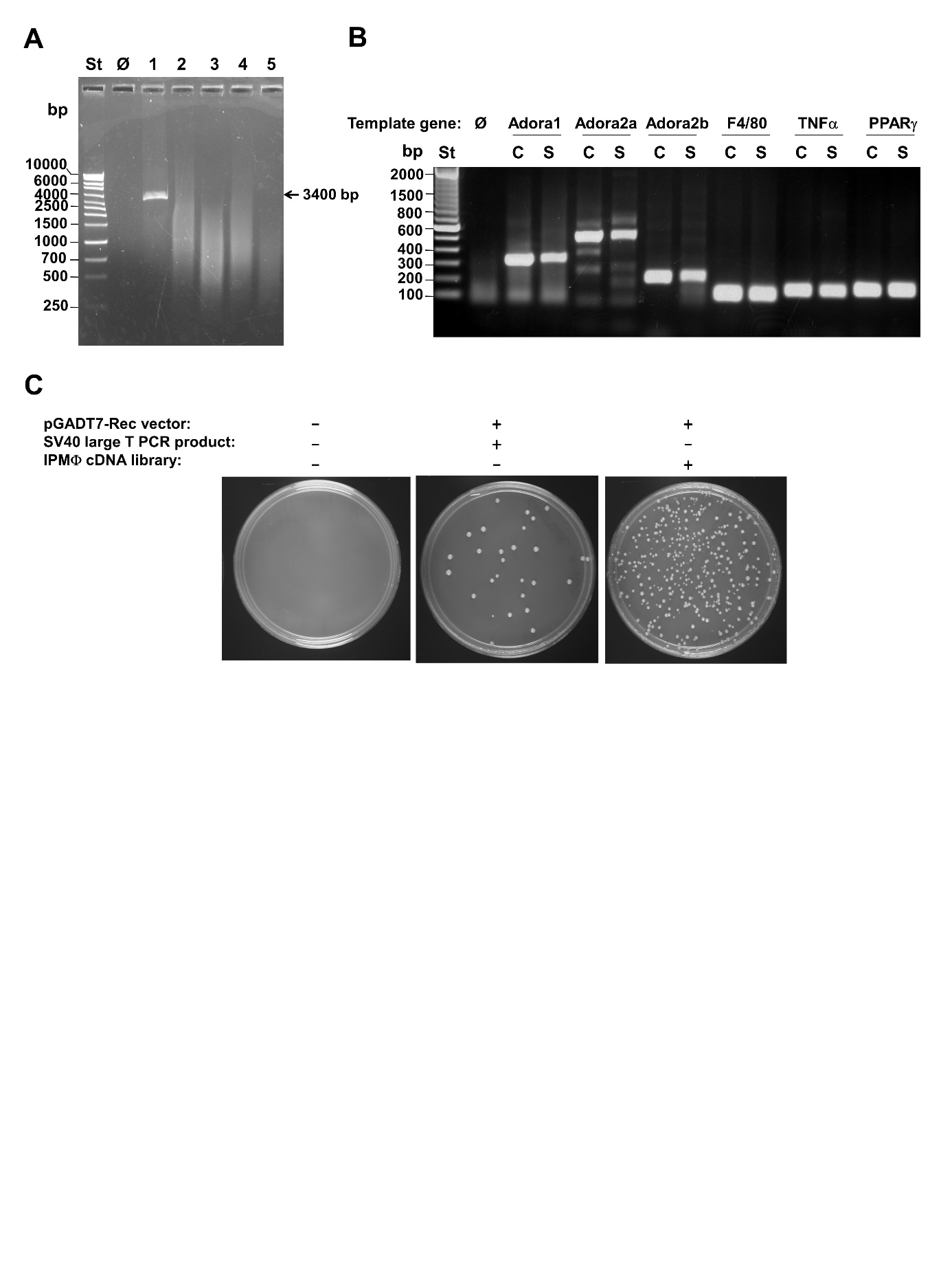
** *Figure S1.* ***Construction of a double-stranded (ds) cDNA library from mouse peritoneal macrophages (IPMΦ) by cloning into the pGADT7-Rec yeast two-hybrid “prey” vector. (A)*** *Two µg of total RNA was isolated from murine IPMΦs and the cDNA was reverse transcribed and amplified using a long range polymerase chain reaction. Lane 1: The PCR product of trypsin inhibitor gene from Calf thymus DNA (100 ng) was used as a positive control; lane 2: ds PCR product from control mouse liver cDNA template pool; lane 3: ds PCR product from mouse IPMΦ cDNA template before ChromaSpin purification; lane 4: IPMΦ ds cDNA sample after ChromaSpin purification; and lane 5: residual ds cDNA (flow-through of ChromaSpin purification).* ***(B)*** *Quality control of the cDNA library. PCR amplification of six different genes: Adora1, Adora2a, and Adora2b, F4/80 receptor, tumor necrosis factor α (TNFα), and peroxisome proliferator-activated receptor γ (PPARγ) using mouse liver ds cDNA (C) and mouse IPMΦ ds cDNA (S) as templates. Ø denotes no template control; St denotes in (A) 1 kb; in (B) 100 bp DNA ladder. Samples were separated on 1% agarose gel and were visualized by ethidium-bromide staining.* ***(C)*** *2.3 µg ds cDNA library was co-transformed with 4 µg linearized pGADT7-Rec “prey” vector into the Saccharomyces cerevisiae Y187 strain. The efficiency of the recombination was tested on synthetic dropout plates lacking leucine and tryptophan (SD/Leu^-^ Trp^-^). Transformed yeast cells were plated on SD/Leu^-^ Trp^-^ selective media at 100x dilution and were incubated for 3 days at 30 °C. Non-transformed Y187 cells were used as a negative control. In the positive control, 25 ng SV40 large T PCR product and 0.5 µg pGADT7-Rec vector were co-transformed into Y187 yeast cells.*


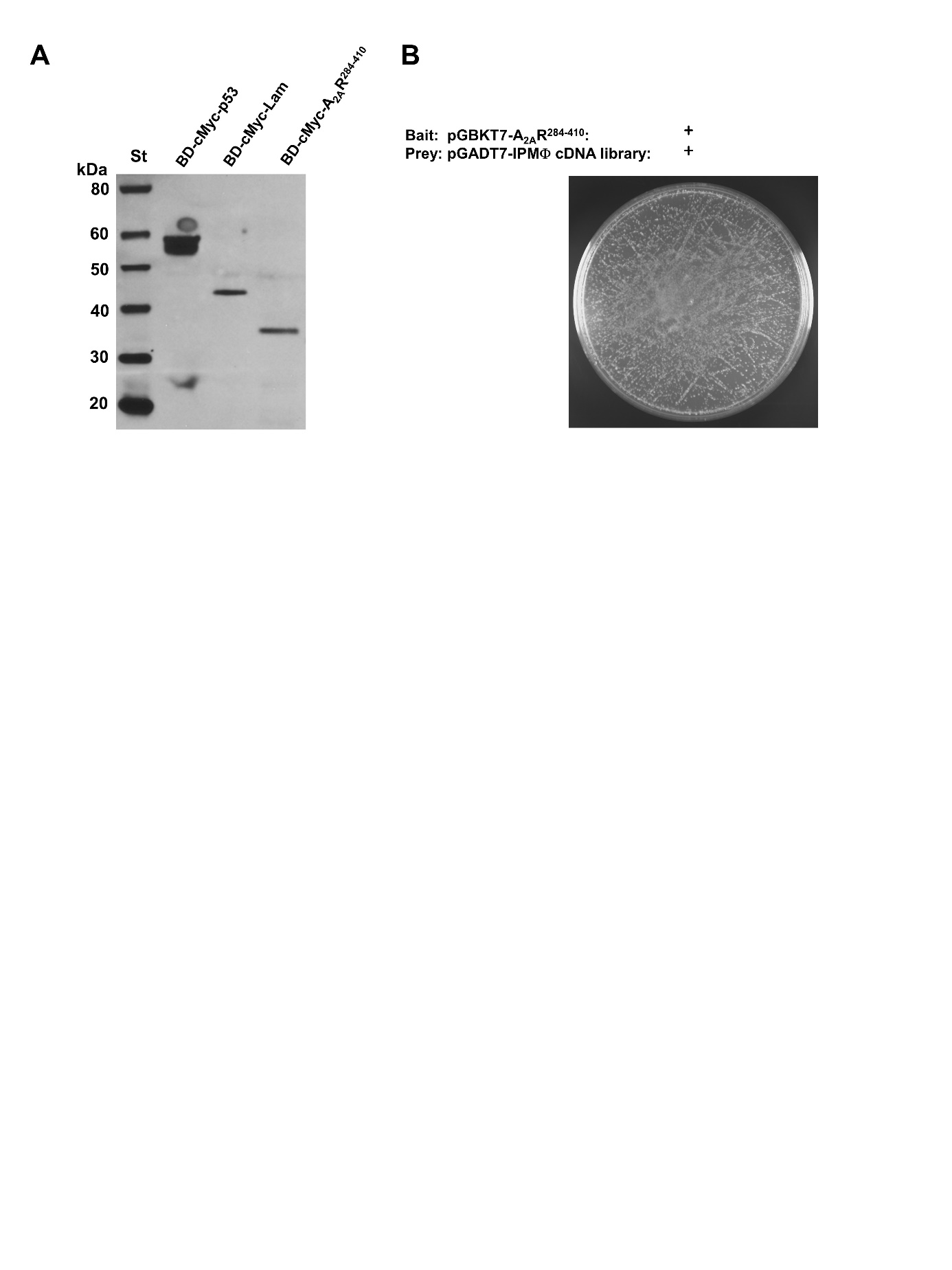
 *Figure S2.* ***Validation of YTH system bait construct and yeast mating.*** *Expression of GAL4 DNA-BD-A_2A_R^284-410^ “bait” protein in yeast and mating with mouse IPMΦ cDNA library containing yeast cells.* ***(A)*** *The expression of the GAL4 DNA-binding domain (BD) fusion protein BD-cMyc-A_2A_R^284-410^ and the controls BD-cMyc-p53, BD-cMyc-Lamin (Lam) was tested using cMyc specific antibodies in yeast Y2H Gold strain, by WB. St denotes Protein MagicMarker.* ***(B)*** *Mating and growing of the BD-cMyc-A_2A_R^284-410^ “bait” expressing Y2H Gold strain with the IPMΦ cDNA library containing Y187 yeast cells. The mated yeast cells were plated on SD/Leu-Trp- selective media in the presence of X-α-Gal (40 µg/ml) and Aureobasidin A (125 ng/ml) and Kanamycin (50 µg/ml) and were incubated for 3 days at 30 °C. A representative plate is shown.*


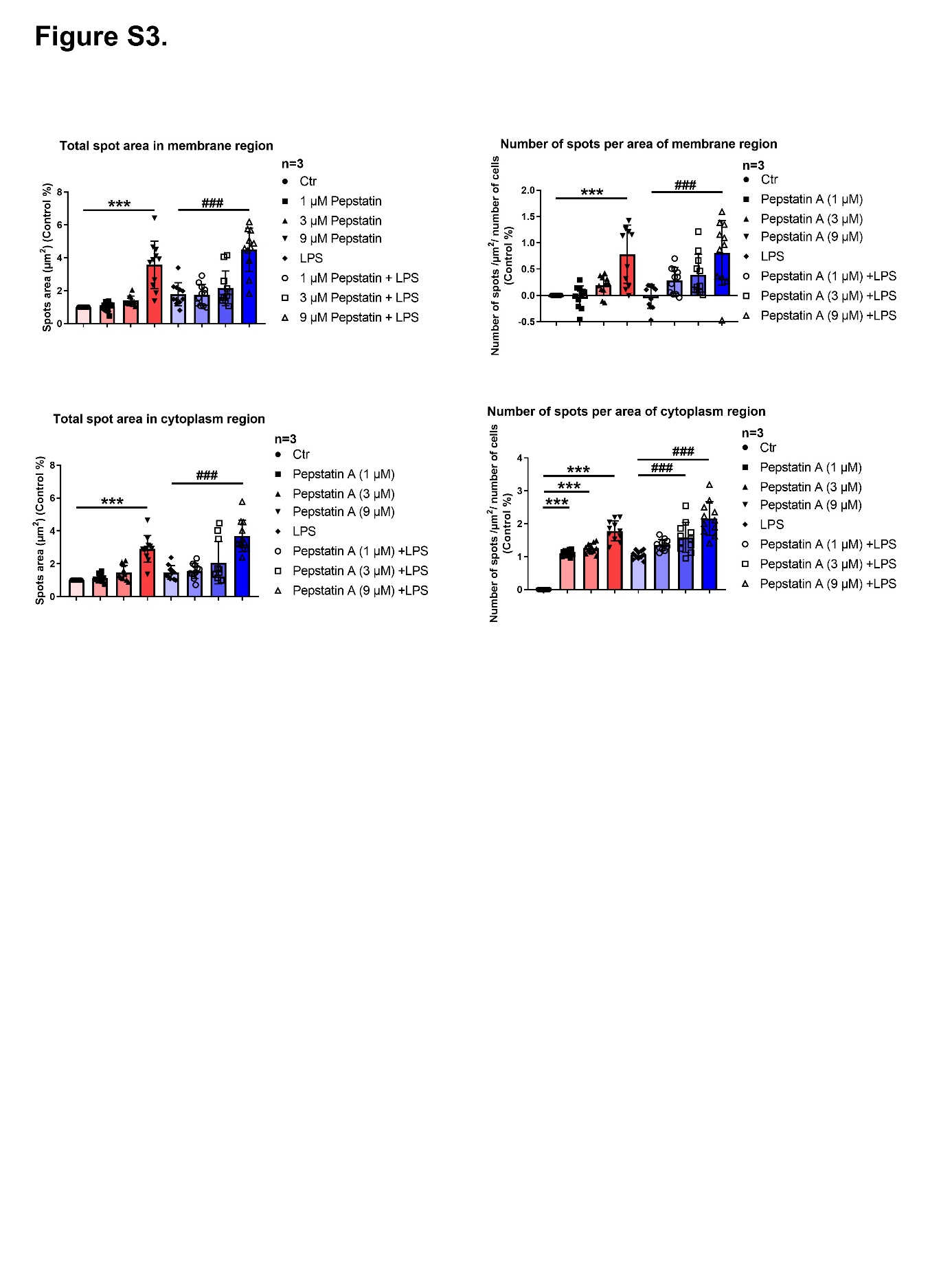


*Figure S3.* ***Pepstatin A penetratin increases A_2A_R expression in mouse macrophages****. The primary data of Figure 4 were analyzed using Harmony 4.8 software (Perkin Elmer, Waltham, MA, USA). Spot Analyses Ready to Made Solution (http://www.perkinelmer.com/product/harmony-4-2-office-hh17000001) was used with custom modifications. Image intensities were rescaled and cells were identified using the DAPI signal. Cellular phenotypes were characterized based on the Alexa488 signal. Cellular features, such as the total spot area and number of spots per area of membrane- and cytoplasm regions, were extracted. Statistical analyses of parallel data sets were made using GraphPad Prism 7 program. The evaluation of the data based on the individual analyses of 2000-3000 different cells is presented as mean ± SD. Values from ANOVA analysis: F=23.15 and P*<*0.001, F=20.16 and P*<*0.001 for Total spot area in membrane-, cytoplasm region, respectively; F=9.982 and P*<*0.0001, F=56.63 and P*<*0.0001 for Number of spots per area of membrane-, cytoplasm region, respectively. ***p < 0.001 vs. control (vehicle-treated); ### p < 0.001 vs. LPS-treated cells.*
